# Cooperative and conformist behavioural preferences predict the dual dimensions of political ideology

**DOI:** 10.1038/s41598-023-31721-6

**Published:** 2023-03-25

**Authors:** Scott Claessens, Chris G. Sibley, Ananish Chaudhuri, Quentin D. Atkinson

**Affiliations:** 1 School of Psychology, University of Auckland, Floor 2, Building 302, 23 Symonds Street, Auckland, 1010 New Zealand; 2 Department of Economics, University of Auckland, Auckland, New Zealand; 3 CESifo, Munich, Germany

**Keywords:** Psychology, Human behaviour, Social evolution

## Abstract

Decades of research suggest that our political differences are best captured by two dimensions of political ideology. The dual evolutionary framework of political ideology predicts that these dimensions should be related to variation in social preferences for cooperation and group conformity. Here, we combine data from a New Zealand survey and a suite of incentivised behavioural tasks (*n* = 991) to test whether cooperative and conformist preferences covary with a pair of widely used measures of the two dimensions of political ideology—Social Dominance Orientation (SDO) and Right Wing Authoritarianism (RWA)—and related policy views. As predicted, we find that cooperative behaviour is negatively related to SDO and economically conservative policy views, while conformist behaviour in the form of social information use is positively related to RWA and socially conservative policy views. However, we did not find the predicted relationships between punitive and rule following behaviours and RWA or socially conservative views, raising questions about the interpretation of punishment and rule following tasks and the nature of authoritarian conformist preferences. These findings reveal how cooperative and conformist preferences that evolved to help us navigate social challenges in our ancestral past continue to track our political differences even today.

## Introduction

Humans differ profoundly in their views on political issues like income redistribution, taxation, welfare, abortion, and religious instruction in schools. With an increasing focus on political polarisation and the consequent challenges of finding common ground on a host of issues^1,2^, understanding the sources of this variation is more pressing than ever.

The extent to which individuals’ political views reflect a predictable ideological structure, and the psychological explanations for ideological differences, are longstanding questions in social psychology that have received renewed attention in recent years^3^. Political ideology is defined as a set of stable, interrelated beliefs and attitudes that organise views on political and social issues^4^. Traditionally, political ideology has been conceptualised as a unidimensional spectrum that varies from liberalism on the left to conservatism on the right^5,6^. Under this standard view, liberals favour equality and social change, whereas conservatives value hierarchy and traditionalism.

However, interdisciplinary research over the past five decades has repeatedly identified two dimensions of political ideology, rather than a single left-right spectrum. In social psychology, studies of human values have revealed two primary value dimensions underlying political ideology. Rokeach^7^ first classified these as *equality* values (i.e., valuing brotherhood and equal opportunity) and *freedom* values (i.e., valuing independence and free choice within groups). Subsequent work elaborated this two-dimensional value framework, referring to *communalism* and *individualism* values^8^, *self-transcendence* and *conservation* values^9^, and *international harmony* and *national strength and order* values^10^. Studies have also uncovered the two-dimensional structure of political attitudes from ratings of dictionary-based “isms”, labelling these attitudinal dimensions as *unmitigated self-interest* and *tradition-oriented religiousness*^11^. In political psychology, researchers have found support for a two-factor model of political ideology which consists of the constructs *social dominance orientation* (SDO), a preference for group-based hierarchy, and *right-wing authoritarianism* (RWA), a preference for authoritarian aggression and submission^12^. More recently, political psychologists have noted the regular clustering of policy views into *economic* views (e.g., views on income redistribution, taxation, and welfare) and *social* views (e.g., views on abortion and religious instruction in schools)^13,14^. Although these constructs are all psychologically distinct, they share many common elements, suggesting that research has independently converged upon two common underlying dimensions of political ideology.

Even for theoretical frameworks that suggest more complicated ideological structures, it can often be shown that two dimensions alone are sufficient to explain the data. For example, Moral Foundations Theory proposes that political ideology is explained by people’s sensitivity to five moral foundations^15^. However, recent work has suggested that these five foundations can be reduced to two primary clusters of moral concerns: *individualising moral foundations*, capturing ethics regarding care and harm, and *binding moral foundations*, capturing ethics regarding ingroup loyalty, deference to authority, and purity. These two primary clusters are related to other two-dimensional approaches to political ideology in predictable ways^16^.

Since political attitudes have a non-trivial heritable component^17^, it is possible that the two dimensions of political ideology have a partly biological basis. Indeed, evolutionary theorists have attempted to explain the existence of the two dimensions in various ways. For example, political egalitarianism has been shown to covary with upper-body strength and physical dominance^18^, whereas authoritarianism and traditionalism have been linked to the behavioural immune system^19^, negativity bias^20^, and adherence to social norms^21^. Moreover, the two dimensions of ideology have distinct psychological correlates. Studies have shown that economic conservatism covaries with agreeableness^22^ and Machiavellianism^23^, while social conservatism covaries with openness to experience^24^ and need for closure^25^. But while these previous theories and studies have illuminated aspects of each dimension separately, they do not attempt to explain the existence of a two-dimensional ideological structure from first principles.

### The dual evolutionary foundations of political ideology

Since politics is fundamentally the process by which humans deal with the myriad conflicts that arise from group living^26,27^, an evolutionary account of the two-dimensional structure of political ideology must be consistent with research on the evolution of human group living. In fact, evidence from anthropology, primatology, and developmental psychology suggests that human group living evolved in two key steps^28–32^. In the first key step, humans became obligate cooperators, responding to resource scarcity by cooperating across wider interdependent networks and sharing the spoils of their cooperation equally. In the second key step, humans became group-minded, delineating their group’s boundaries with cultural markers and adhering to and enforcing group-wide social norms. Throughout the Pleistocene, these two key steps are thought to have paved the way from small kin bands to larger, more complex communities of hunter-gatherers.

These two primary responses to the challenges of human group living—cooperation and group conformity—derived independently from work in political psychology, offer a first principles explanation for the two foundational dimensions of political ideology that have been repeatedly uncovered in the social sciences^5^. The first dimension is broadly concerned with the trade-off between cooperative egalitarianism and competitive hierarchy. For example, equality and self-transcendence values reflect altruistic egalitarian motives, whereas unmitigated self-interest and SDO reflect competitive and selfish motives that promote inequality. The second dimension is broadly concerned with the trade-off between individualism and group conformity. For example, freedom and individualism values reflect independence from social control, whereas binding moral foundations and RWA reflect conformity to group-wide social norms. Under this account, these domains of cooperation and group conformity are predicted to shape human political psychology, not just in Western liberal democracies, but also in non-Western and small-scale societies.

This dual evolutionary theory^5^ brings together and intersects with a diverse array of material, combining independent lines of evidence from multiple disciplines. But more work is required to develop and test the theory. In this paper, we provide the first test of one central prediction of the theory: that individual differences in general behavioural preferences for cooperation and group conformity can explain why people vary along the two dimensions of political ideology^5^. We define cooperative behaviour as behaviour that provides a benefit to another individual at a personal cost^33^ and conformist behaviour as reliance on socially acquired information and adherence to and enforcement of social norms within cultural groups. Individual differences in behavioural preferences for cooperation and group conformity are thought to arise from a combination of biological predispositions and responses to environmental conditions. Based on this central prediction, an increased general behavioural preference for cooperation beyond close kin results in greater support for policies that promote large-scale impersonal cooperation and a more egalitarian distribution of resources, such as income redistribution, taxation, welfare, and pro-environmentalism. An increased general behavioural preference for group conformity results in greater support for policies that promote in-group conformity, norm enforcement, and the interests of the in-group, such as religious instruction in schools, capital punishment, and military spending.

### Previous research in behavioural economics

If general behavioural preferences for cooperation and group conformity do underlie differences in political opinion in modern humans, such differences should be manifest in preferences measured by domain-general incentivised behavioural tasks that involve decision-making with salient monetary rewards. Such tasks allow researchers to study variation in enduring social preferences without succumbing to issues of social desirability and experimenter demand that plague self-report methods^34–36^.

Previous research has studied the covariation between behaviour in incentivised tasks and the two dimensions of political ideology. A number of studies find support for an association between cooperative preferences and SDO, an ideological measure of preferences for hierarchy and anti-egalitarianism^37,38^. A recent meta-analysis of the links between personality and prosocial behaviour found that cooperative behaviour in a number of social dilemma games that allow exploitation of others was negatively correlated with SDO^39^. In contrast, other research has failed to find a relationship between SDO and cooperative behaviour in the Prisoner’s Dilemma Game, in which two players choose whether to cooperate or defect^37,40^, or the Stag Hunt Game, in which two players must coordinate on one of multiple equilibrium strategies^37^. These findings suggest that SDO may relate to particular affordances of individual games (e.g., exploitation^39^) rather than to a general cooperative preference that applies across situations with different payoff structures, as predicted by the dual evolutionary framework.

While self-report studies have found correlations between socially conservative and conformist attitudes^41,42^, behavioural studies exploring the relationship between political ideology and general behavioural conformist preferences are more limited. Several studies have found a relationship between conformist social learning behaviour and collectivist values^43,44^. Collectivist values are positively correlated with RWA, an ideological measure of authoritarian attitudes^45^. In contrast, another study found that norm-enforcing punishment behaviour in a collective action game did not differ between Republicans and Democrats, although these authors did not include measures of political ideology^46^. Much like the work relating cooperative behaviour to political ideology, these behavioural studies are limited by only focusing on one or two incentivised behavioural tasks and single measures of political ideology.

### The current study

Here, we extend prior work by combining a large suite of incentivised behavioural tasks with comprehensive survey data to test whether and to what extent cooperative and conformist behavioural preferences covary with measures of the two dimensions of political ideology. Across two pre-registered studies, we asked participants to complete a series of incentivised behavioural tasks measuring cooperative preferences, such as trust, altruism, and collective action, and conformist preferences, such as norm-enforcing punishment, social information use, and rule following. In Study 1, we used a battery of one-shot anonymous economic games, selected because they have previously been shown to capture two dimensions of behaviour, one reflecting a “cooperative phenotype” and another labelled “norm-enforcing punishment”^47^. In Study 2, we extended this suite of games to include incentivised tasks capturing two further elements of conformist behaviour: social information use and rule following. In both studies, we used abstract, context-free behavioural tasks to best capture the general context-free behavioural preferences for cooperation and group conformity posited by our theory.

Across these studies, we related cooperative and conformist preferences to SDO and RWA, two widely used measures that map onto the hypothesised dimensions of political ideology^12^ and have been shown to predict policy views, prejudice, and political party support^12,48,49^. However, useful though they are, these measures are not perfect proxies of the two latent dimensions of political ideology proposed by the dual evolutionary framework^5^. For example, SDO items refer to between-group rather than interpersonal competition^50^ and the relationship between RWA and conservatism is context-dependent^51^. To generalise our findings beyond these idiosyncratic measures, we also related cooperative and conformist behaviour to a host of locally relevant economic and social policy views, respectively. By linking behaviour in these abstract tasks to a broad range of self-reported political attitudes and policy views, we aim to identify whether and to what extent cooperative and conformist preferences covary with the dual dimensions of political ideology.

## Study 1

### Methods

#### Participants and sampling

Participants for this study were sampled from the ongoing longitudinal New Zealand Attitudes and Values Study^52^. This participant pool is a representative sample of the New Zealand population, contacted through random draws from the New Zealand electoral roll. From a sample frame of 3345 participants (see Supplementary Methods), we successfully contacted 2731 about the study. Participants were contacted initially by phone and then, if they agreed to participate, over email in the days leading up to their allocated study session. 1740 participants either dropped out of the study, spent less than 5 min or more than 50 min on the games, or did not have SDO or RWA scores. This resulted in a final sample of 991 participants, which exceeded our apriori power analysis (see Supplementary Methods). While this final sample was likely not representative of the New Zealand population, it was highly diverse both demographically (661 females; age *M* = 51.8 years, *SD* = 12.1 years; see Supplementary Fig. S1 for further sample characteristics) and, crucially for our study, politically (left-wing Labour Party supporters = 360, right-wing National Party supporters = 330, other or not declared = 301). Because this is an ongoing longitudinal study, ethical concerns prevent us from making the final dataset publicly available.

#### Materials

##### New Zealand Attitudes and Values Survey measures

We took our main self-report variables from the 10th wave of the New Zealand Attitudes and Values Study^52^, an annual longitudinal survey completed by New Zealand participants. The main self-report variables included SDO, RWA, age, gender, ethnicity, education level, socio-economic status, local deprivation, and religiosity. SDO and RWA scores were both mean scores of six separate Likert-scale items (1 = strongly disagree, 7 = strongly agree). These sets of six items were initially chosen for the New Zealand Attitudes and Values Study based on an exploratory factor analysis including all the items from the previously validated 16-item SDO scale^53^ and 30-item RWA scale^54^, choosing a balanced set of pro-trait and con-trait items that loaded strongly onto their respective factor and did not cross-load onto the other factor (see Supplementary Table S1 for factor loadings on a separate validation sample). Additional items on policy views were taken from earlier waves of the New Zealand Attitudes and Values Study. See Supplementary Table S2 for full list of self-report items.

##### Battery of economic games

Participants completed eight economic games, conducted online in real-time using oTree software (code available at https://osf.io/dwx8g/)^55,56^. In an attempt to replicate prior work, these games are largely identical to a previous study^47^.

We included three cooperation games in which individuals decided whether to incur a personal cost to benefit another player:

- *Dictator Game*. Player A is given 100 points. They must decide how many of these points to transfer to Player B. Player A keeps the remaining points. Player B is passive in the interaction.
- *Trust Game*. Players A and B both start with 50 points. First, Player A decides whether or not to transfer all 50 points to Player B, in the knowledge that the transferred amount will be tripled to 150 points. If Player A transfers, Player B now has 200 points. Player B must then decide to transfer 0–150 points back to Player A.
- *Public Goods Game*. Four players begin with 100 points each. They can contribute 0–100 points into a shared group project. All four decisions are made simultaneously, and then the amount in the group project is doubled and distributed evenly between all four players. Each player ends the game with their share from the group project, plus the points they initially refrained from contributing.

We also included three punishment games, in which individuals decide whether to incur a personal cost to punish another player for their decisions:

- *Ultimatum Game*. Player A starts with 100 points, and Player B starts with nothing. Player A must decide how many points to transfer to Player B. However, Player B simultaneously specifies their ‘minimum acceptable offer’: namely, the lowest transfer from Player A that they will accept. If Player A’s transfer amount is lower than this minimum acceptable offer, both players end the game with 0 points. Otherwise, Player B receives the transfer amount, and Player A keeps the remaining points.
- *Third-party Punishment Game*. Players A, B, and C all start with 100 points. First, Player A decides whether to ‘take’ from Player B. If Player A takes, Player B loses 50 points and Player A gains 30 points (taking is inefficient). If Player A takes, Player C can then pay 0–20 points to remove points from Player A. Each paid point removes 5 points from Player A. Player B is passive in the interaction.
- *Second-party Punishment Game*. Players A and B start with 100 points. This game has two stages: the transfer stage, and the penalty stage. In the transfer stage, each player decides whether to transfer 30 points to the other player. Any transferred points are doubled before the other player receives them. Decisions are made simultaneously. The transfer stage follows the payoff matrix of a Prisoner’s Dilemma. Then, in the penalty phase, both players can pay 0–10 points to remove points from the other player, depending on their decision in the transfer stage. Each paid point removes 5 points from the other player.

We replaced the destructive All-Pay Auction Game used in prior work^47^ with two coordination games: a version of the Stag Hunt Game, in which players must coordinate on one of multiple equilibria, with one of those equilibria dominating the others in terms of payoffs; and the Stag Hunt Game with Punishment, in which players may punish others for not choosing the strategy commensurate with the payoff-dominant equilibrium. We added these two games to study whether the general preferences for cooperation and punishment extended to coordination problems that do not involve exploitation of others and are arguably better models for the real-world cooperative dilemmas faced by our human ancestors^28^:

- *Stag Hunt Game*. Four players begin with 50 points each. They can either contribute 30 points into a shared group project or contribute nothing. Any points in the group project will be doubled and distributed evenly between all players, *but only if all players contribute*. Otherwise, the points in the group project will be lost. Decisions are made simultaneously. Each player ends the game with their share from the group project, plus the points they initially refrained from contributing.
- *Stag Hunt Game with Punishment*. Players A and B start with 100 points. This game has two stages: the transfer stage, and the penalty stage. In the transfer stage, each player decides whether to transfer 30 points to a group project. Any points in the group project will be doubled and distributed evenly between both players, *but only if both players contribute*. Otherwise, the points in the group project will be lost. Decisions are made simultaneously. The transfer stage follows the payoff matrix of a Stag Hunt. Then, in the penalty phase, both players can pay 0–10 points to remove points from the other player, depending on their decision in the transfer stage. Each paid point removes 5 points from the other player.

All games involved one-shot decisions between multiple anonymous players. Such one-shot anonymous games remove the well-documented effects of communication, signalling, and/or reputation building on behaviour^34,57^ and avoid potential confounds arising from repeated play, given that repeated game play may result in “super-games” and consequent cooperation even in finite repetitions^58^. The strategy method, in which participants provide responses for all possible roles in the games, was used to measure responses. This approach avoids feedback from other players, allowing us to make claims about cooperative preferences. The strategy method has been shown to elicit similar behaviour to sequential play^59^.

Participants played for points, which were converted to New Zealand dollars (1 point = $0.035). Players were matched ex-post after making decisions in each of the eight games. One role per game was randomly chosen as the payoff-relevant decision role. Decisions were then matched randomly to the decisions of other players in the session to determine earnings. Earnings were accumulated across all games to ensure equivalent reward salience throughout the session and to avoid suggesting to participants that some of their decisions were hypothetical and not being played for real money. To avoid potential carry-over and wealth effects introduced by this payment schedule, feedback on earnings was provided at the end of the session and not between games. We conducted sessions in real-time to ensure that participants could be paid immediately at the end of the session. This real-time interaction also ensured that participants were aware that they were interacting with others in a contemporaneous manner and did not feel as if they were playing with computerised partners, which can elicit very different patterns of behaviour^60,61^.

#### Procedure

Data collection for Study 1 was conducted between 18th February and 25th July 2019. Study sessions contained between 14 and 97 participants, and were conducted on midweek evenings (see Supplementary Methods). Participants knew that they were playing with others recruited from the New Zealand Attitudes and Values Study, but were not aware of how many people were present in any particular session. Participants learned that they would be playing for points that would be later converted to currency, that they would be matched in real-time with other participants from the New Zealand Attitudes and Values Study, and that every “task” would count towards their final bonus payment.

The eight economic games were presented in a random order. For each game, participants read the instructions and answered a comprehension question about the game. Responses to the comprehension questions suggested that participants understood the structure of all of the games (Supplementary Table S3). Participants then provided responses for all possible roles in the game, following the strategy method. After all the games, participants entered a waiting lobby where they waited for other participants in the session to complete the games. Once all participants in the session arrived at the waiting lobby, the software calculated payoffs for each game by randomly matching individuals within a session. The accumulated payoffs across all eight games determined the final overall payoff.

Participants were paid a fixed $20 NZD show-up fee, which ensured at least the living wage payment for a 50 min study session. This show-up fee was important to make sure that no participant felt disappointed with the study, thereby leading them to drop out of the larger New Zealand Attitudes and Values Study. In addition, participants could earn a bonus payment of between $10 and 35 (*M* = $25.11, *SD* = $2.51) depending on the decisions made by them and their peers. While these bonus payments were comparable in size to the show-up fee, previous work has shown that even relatively low stakes do not cause marked changes in behaviour^62^. Nevertheless, it should be noted that the absolute value of our bonus payment is higher than that provided in many behavioural economics studies. On average, participants took 22 min to complete the eight economic games (*SD* = 7.36 min, range = 6–47 min).

#### Pre-registration

We pre-registered three hypotheses on the Open Science Framework on 19th November 2018 (https://osf.io/dwx8g/). First, we hypothesised that a two-factor structure of cooperation and punishment would emerge from the suite of economic games, replicating previous work^47^. Second, we hypothesised that SDO would negatively predict the cooperation factor. Third, we hypothesised that RWA would positively predict the punishment factor.

#### Statistical analysis

Our pre-registered analyses proceeded in two stages. In the first stage, we used a comprehensive set of statistical tests (correlations, principal components analyses, and confirmatory factor analyses) to determine the factor structure of variation in gameplay. We expected that two factors would emerge from and be supported by the data: cooperation and punishment. In the second stage, we used structural equation modelling to determine the extent to which these cooperation and punishment latent variables could be predicted by SDO and RWA. We also conducted additional exploratory analyses that were not pre-registered: (1) analyses regressing SDO and RWA onto individual game decisions, and (2) structural equation models regressing policy views onto the cooperation and punishment latent variables. For all confirmatory factor models and structural equation models, we controlled for game comprehension by including the proportion of correct comprehension questions as a predictor of all game decisions, and fitted the models using diagonally weighted least squares estimation.

All analyses were conducted in R Version 4.0.2^63^. We used the *psych* package^64^ for correlations and principal components analyses, and the *lavaan* package^65^ for confirmatory factor analyses and structural equation modelling. Figures were created with the *ggplot2*^66^ and *cowplot*^67^ packages, and the manuscript was reproducibly generated with the *drake*^68^ and *papaja*^69^ packages. Code to reproduce all analyses can be found on the Open Science Framework: https://osf.io/dwx8g/^55^.

#### Ethics statement

Ethical approval was granted by the University of Auckland Human Participants Ethics Committee (ref: 021666). The study was performed in accordance with all the relevant guidelines and regulations. Informed consent was obtained from all participants prior to the study.

### Results

**Figure 1:**
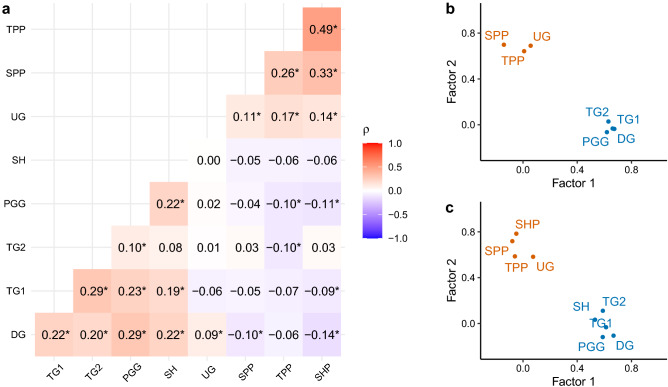
Factor structure of economic games. (**a**) Pairwise Spearman’s rank correlations for game decisions. * indicates significant correlations with Benjamini-Hochberg corrected *p* values (*p* $< 0.050$). (**b**) Factor loadings from a principal components analysis with only the game decisions from previous work^47^. (**c**) Factor loadings from an extended principal components analysis including coordination games. DG = Dictator Game, TG1 = Trust Game (Give), TG2 = Trust Game (Return), PGG = Public Goods Game, SH = Stag Hunt Game, UG = Ultimatum Game (Minimum Acceptable Offer), SPP = Second-Party Punishment Game (Punish Defector), TPP = Third-Party Punishment Game (Punish), SHP = Stag Hunt Game with Punishment (Punish Defector).

Before examining the relationship between gameplay and ideology, we first tested whether behaviour across the games shows a two-dimensional structure in line with our pre-registered expectations. Distributions of game decisions are reported as histograms in Supplementary Fig. S2. Pairwise Spearman’s rank correlations between game decisions, accounting for multiple comparisons using Benjamini-Hochberg corrected *p* values, are visualised in Fig. 1a. Replicating previous work^47^, we found significant positive correlations between most cooperation decisions ($\rho$ = 0.08–0.29), significant positive correlations between all punishment decisions ($\rho$ = 0.11–0.49), and non-significant or negative correlations between most cooperation and punishment decisions. Principal components analyses suggested that these patterns of covariation could be explained by two underlying principal components: cooperation and punishment. When including only games from previous work, principal components analysis using orthogonal varimax rotation supported a two-dimensional solution, based on the point of inflexion in the scree plot and the number of principal components with eigenvalues above one^70,71^ (Supplementary Fig. S3a). This analysis revealed that cooperative behaviour loads highly onto one principal component and punitive behaviour loads highly onto a second distinct principal component (Fig. 1b). Together, these factors explained 44% of the variance in game decisions. The two-dimensional structure held when adding our novel coordination games (Fig. 1c and Supplementary Fig. S3b; 41% variance explained). This structure was also supported by exploratory parallel analysis, which independently proposed a two-dimensional solution (Supplementary Fig. S4), and confirmatory factor analyses, which were robust to the inclusion of method factors (see Supplementary Results).

Having confirmed the existence of a two-dimensional structure underlying gameplay, we next fitted pre-registered structural equation models to test the relationships between the two latent variables and political ideology. For reference, previous work has shown that effect sizes for relationships between economic game behaviour and self-reported attitudes tend to vary from very small to medium^39^ (0.00 $\le$ *r* $\le$ 0.26).

**Figure 2:**
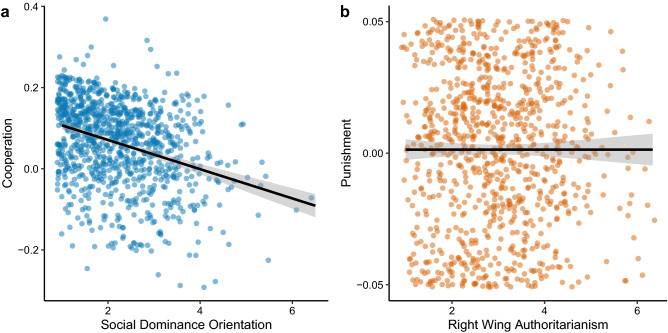
Relationships between model-predicted latent variable scores for cooperation and punishment and the two dimensions of political ideology. (**a**) Social Dominance Orientation (mean score) is negatively related to the cooperation latent variable. (**b**) Right Wing Authoritarianism (mean score) is unrelated to the punishment latent variable. Full lines are predictions from least-squares linear regressions without demographic covariates and shaded areas are 95% confidence intervals. Note that the latent variable scores are not raw data, but rather model predictions that are estimated with error that is not visualised in the figure.

We simultaneously regressed the cooperation and punishment latent variables onto mean scores of six Likert-scale items for SDO (Cronbach’s $\alpha$ = 0.80, $\omega$ = 0.84) and RWA ($\alpha$ = 0.73, $\omega$ = 0.74). In line with our second pre-registered hypothesis, we found that SDO significantly negatively predicted the cooperation latent variable with a small-to-medium effect size (standardised $\beta$ = $-0.24$, unstandardised *b* = $-0.04$, 95% confidence interval [$-0.05$, $-0.03$], *p* $< 0.001$, semi-partial *r* = 0.25; Fig. 2a). SDO also significantly positively predicted the punishment latent variable with a small effect size, which we did not hypothesise ($\beta$ = 0.13, *b* = 0.005, 95% CI [0.002, 0.008], *p* $< 0.001$, semi-partial *r* = 0.13), though the magnitude of this association was not as strong as with the cooperation latent variable (difference in absolute unstandardized coefficients = $-0.03$, SE = 0.006, p $< 0.001$). In contrast with our third pre-registered hypothesis, we found no significant relationship between RWA and the punishment latent variable ($\beta$ = $-0.01$, *b* = 0.000, 95% CI [$-0.002$, 0.002], *p* = 0.848, semi-partial *r* = 0.00; Fig. 2b). RWA was also unrelated to the cooperation latent variable ($\beta$ = $-0.02$, *b* = 0.00, 95% CI [$-0.01$, 0.01], *p* = 0.472, semi-partial *r* = 0.06). This pattern of results was robust to method bias (Supplementary Results) and held when additionally controlling for age, gender, ethnicity, education, socio-economic status, local deprivation, and religiosity (Supplementary Table S4).

To further investigate the relationships between political ideology and gameplay, we conducted a series of exploratory linear regressions with participants’ mean SDO and RWA scores as outcome variables and individual game decisions as predictors, accounting for multiple comparisons and statistically controlling for demographics and game comprehension. We found that most cooperative decisions were negatively associated with SDO (Supplementary Fig. S5). Holding all other variables constant, individuals with higher SDO scores gave less in the Dictator Game, returned less in the Trust Game, contributed less in the Public Goods Game, did not coordinate in the Stag Hunt Game, offered less in the Ultimatum Game, and were more likely to take in the Third-Party Punishment Game. The effect sizes for these relationships were small, but consistent across games (semi-partial *r* = 0.09–0.11). Contrary to our predictions, SDO was also positively related to both “social” and “antisocial” punishment decisions in the Second-Party Punishment Game and Stag Hunt Game with Punishment (i.e., costly punishment of defectors and cooperators, respectively). Fewer individual game decisions predicted RWA (Supplementary Fig. S6). Holding all other variables constant, individuals with higher RWA scores were more likely to take in the Third-Party Punishment Game and engaged in greater anti-social punishment in the Second-Party Punishment Game and the Stag Hunt Game with Punishment.

**Figure 3:**
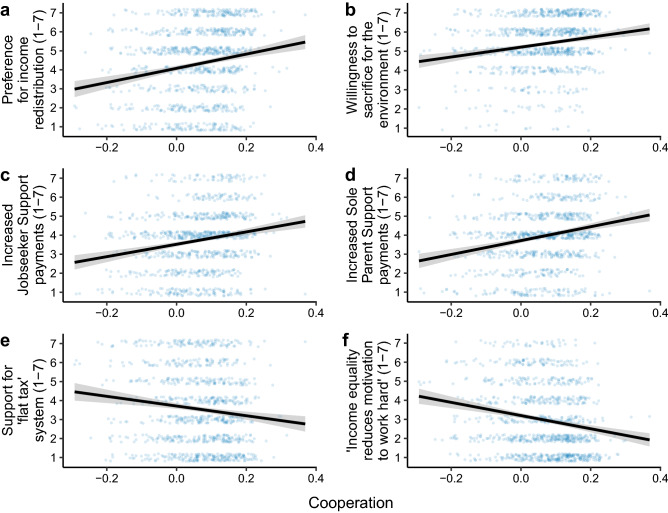
The cooperation latent variable predicts a host of economically progressive views. For every Likert scale, 7 indicates increased support for the policy or agreement with the statement, whereas 1 indicates reduced support for the policy or disagreement with the statement. For visualisation ease, regression lines and 95% confidence interval shaded areas are predictions from least-squares linear regressions without demographic covariates. Note that the latent variable scores are not raw data, but rather model predictions that are estimated with error that is not visualised in the figure.

The cooperation latent variable also reliably predicted economically progressive views across a wide range of political issues (Fig. 3; Supplementary Tables S5–S13). Controlling for the punishment latent variable, game comprehension, and demographics, exploratory structural equation modelling revealed that the cooperation latent variable positively predicted preferences for income redistribution (*b* = 1.08, 95% CI [0.48, 1.68], *p* $< 0.001$, semi-partial *r* = 0.12), making sacrifices for the environment (*b* = 1.12, 95% CI [0.35, 1.89], *p* = 0.004, semi-partial *r* = 0.12), increased payments to those receiving Jobseeker Support (*b* = 1.31, 95% CI [0.63, 1.98], *p* $< 0.001$, semi-partial *r* = 0.16), and increased payments to those receiving Sole Parent Support (*b* = 1.40, 95% CI [0.72, 2.08], *p* $< 0.001$, semi-partial *r* = 0.16). Cooperation was also negatively related to support for an economically conservative “flat tax” where everyone pays the same percentage of tax on their income regardless of their wealth (*b* = $-0.70$, 95% CI [$-1.33$, $-0.06$], *p* = 0.032, semi-partial *r* = 0.09) and the belief that people would be less motivated to work hard if incomes were equal (*b* = − 1.33, 95% CI [$-1.94$, $-0.73$], *p* $< 0.001$, semi-partial *r* = 0.15). Though these individual effect sizes are small, the cooperation latent variable explains a similar proportion of variance in economic policy views to other demographic variables (Supplementary Fig. S7). Moreover, the effect size was larger when combining progressive economic policy views into a single latent variable: the cooperation latent variable was positively related to this latent variable with a medium effect size ($\beta$ = 0.29, *b* = 1.33, 95% CI [0.84, 1.81], *p* $< 0.001$, semi-partial *r* = 0.29).

By contrast, the punishment latent variable did not predict social views like support for same-sex marriage (*b* = 0.12, 95% CI [$-2.60$, 2.84], *p* = 0.931, semi-partial *r* = 0.01), euthanasia (*b* = 0.92, 95% CI [$-1.69$, 3.54], *p* = 0.489, semi-partial *r* = 0.03), or abortion (*b* = 1.05, 95% CI [$-1.28$, 3.37], *p* = 0.377, semi-partial *r* = 0.04). The punishment latent variable was also unrelated to a latent variable that captured progressive views on all these social policies combined ($\beta$ = $-0.04$, *b* = $-1.21$, 95% CI [$-4.10$, 1.69], *p* = 0.413, semi-partial *r* = 0.04).

### Discussion

We found that behaviour across a large suite of incentivised economic games can be captured by two underlying general preferences: cooperation and punishment. This finding replicates previous work^47,72^ and demonstrates that the cooperative phenotype generalises to an English-speaking sample outside the United States and Europe. In addition, the unique combination of substantial monetary stakes, real-time interactive gameplay, and additional coordination games deployed in our study indicates that the cooperative phenotype emerges with methodologies that may more reliably track real-world social interactions and with games that do not involve exploitation of others^39^.

As predicted by the dual evolutionary framework of political ideology^5^, we found that the cooperative phenotype captured by our economic games was negatively related to SDO, with an effect size comparable to that found in a recent meta-analysis of personality traits and economic game behaviour^39^. When we separately explored individual economic games, we found that the negative correlation between SDO and cooperation held for most cooperative decisions, corroborating previous work identifying negative correlations between SDO and cooperative behaviour in individual games^37–39,73^. Further exploratory analyses revealed that the cooperative phenotype was related to economically progressive views across a wide range of policy issues, including environmentalism, welfare, taxation, and income redistribution. While the effect sizes for these associations were consistently small, the cooperation latent variable explained a comparable proportion of variance to other demographic variables known to predict economically progressive policy views, such as age, gender, and education^13,74^, and the effects persisted when controlling for these variables.

We did not find the predicted association between the punishment latent variable and RWA, nor did we find any link between the punishment latent variable and socially conservative policy views. One explanation for this lack of relationship is that while authoritarians might desire the sanctioning of norm violators, they would rather this punishment come from a third-party institution that they perceive to be legitimate than from an interpersonal encounter. Indeed, RWA items refer to sanctioning from strong leaders or governments, but rarely from individuals themselves^12^. Another explanation is that the punishment games used in this study do not reflect general group conformist preferences as expected. As has been suggested previously^75,76^, punishment in economic games may more closely track a competitive motive to increase one’s relative payoffs over others, especially when the economic games are one-shot games with anonymous others in which there is no possibility for signalling, reputation building, or future behaviour modification.

In light of these concerns about the use of one-shot punishment games as a proxy for general group conformist preferences, we conducted a second study in which we introduced two new tasks intended to measure two types of conformist preferences. The first task is designed to measure conformity in the form of rule following, while the second task is designed to measure conformity in the form of social information use. In Study 2, we re-sampled the same individuals from Study 1 for a second wave of data collection, and included these new tasks to further investigate the nature and scope of any relationship between conformist preferences and RWA and social policy views.

## Study 2

### Methods

#### Participants

In Study 2, we attempted to contact every participant who had completed Study 1 and had not since withdrawn from our study or the larger New Zealand Attitudes and Values Study. This resulted in a sample frame with 997 participants. Of the participants we emailed, 636 participants completed the second wave of data collection (64% retention rate). 27 of these participants did not complete the study in time or did not have RWA scores, resulting in a final sample of 609 participants (411 females; age *M* = 51.0 years, *SD* = 12.5 years).

#### Materials

The materials for Study 2 were mostly identical to Study 1. As in Study 1, eight incentivised behavioural tasks were conducted online in real-time using oTree software (code available at https://osf.io/dwx8g/)^55,56^. The strategy method was used to elicit responses in all possible roles, and participants played for points which were converted to New Zealand dollars at the same rate as in Study 1 (1 point = $0.035). To collect data continuously alongside the longitudinal New Zealand Attitudes and Values Study, the six tasks from previous work^47^—the Dictator Game, Trust Game, Public Goods Game, Ultimatum Game, Third-Party Punishment Game, and Second-Party Punishment Game—were measured again (see Supplementary Table S14 for test-retest reliability). However, in Study 2, the coordination games were replaced with two different tasks.

The first task was the Rule Following Task^77^, a task designed to capture individual differences in conformity in the form of rule following. In this task, participants are required to place 30 balls, sequentially, into one of two buckets. Each ball placed into Bucket A earns the participant one point, while each ball placed into Bucket B earns the participant two points (randomly counterbalanced). Before making any decisions, participants are shown on the screen the text: “the rule is to place the balls into Bucket A”. No explanation is provided as to why this is the rule or why participants should follow the rule, which makes them worse off in monetary terms. There is no penalty to participants who break the rule. Our measure of rule following is how often participants follow the rule by placing the balls into Bucket A.

The second task was the Berlin Estimate AdjuStment Task (BEAST)^44^, a task designed to measure individual differences in conformity in the form of social information use. In this task, over five rounds, participants are required to report the number of animals present in briefly displayed images (see Supplementary Fig. S8). There are two parts to each round. In the first part, the image is displayed for six seconds and participants give their initial estimate of the number of animals ($E_1$). In the second part, participants are then made aware of a previous participant’s estimate for the same image ($s$) and then give a second estimate ($E_2$). Previous participants’ estimates are determined by searching a database of real estimates from 100 previous participants sampled in a previous study^44^. The computer searches for an estimate in this database that is (1) in the direction of the true answer (e.g., if the participant guesses 50 and the true answer is 60, social information will be higher than 50) and (2) deviates from the participant’s first estimate according to a parameter $\Delta$: $s = E_1 * (1 \pm \Delta )$. Following the original paper, this $\Delta$ parameter varied across the five rounds, taking values of 0.25, 0.15, 0.20, 0.15, and 0.25, respectively. Participants were paid for their accuracy following the approach in the original paper^44^, by randomly choosing a round and determining the distance between their estimate and the correct answer: 100 points – (distance * 5).

Based on participants’ estimate in the first part, estimate in the second part, and the social information they were provided, we calculate a score for each of the five rounds using the following equation from previous work^44^:

$$\begin{aligned} \text {BEAST score}=\frac{E_2 - E_1}{s - E_1} \end{aligned}$$

In keeping with previous work^44^, we remove cases where (1) participants moved their second estimate away from the social information, (2) where participants moved their second estimate further than the social information, or (3) participants ran out of time to make their estimate (8% of cases). The BEAST score varies between 0 and 1, where 0 implies that the participant did not shift their estimate after receiving social information and 1 implies that the participant shifted their estimate completely towards the social information. This is our measure of social information use.

#### Procedure

Data collection for Study 2 was conducted between 19th October and 11th November 2020 with weekly staggered recruitment. Study sessions contained between 30 and 130 participants. Sessions proceeded in a similar fashion to Study 1, except that the six economic games were presented in a random order first, and then the new Rule Following Task and BEAST were presented in a random order at the end of the session. As in Study 1, participants in a session were then randomly matched in real-time following the eight tasks and the computer determined bonus payments.

Participants were paid a fixed $20 NZD show-up fee, plus a bonus payment of between $10 and 35 (*M* = $21.41, *SD* = $2.63) depending on the decisions of themselves and others. On average, participants took 24 min to complete the eight tasks (*SD* = 7.52 min, range = 9–49 min).

#### Pre-registration

We pre-registered two hypotheses on the Open Science Framework on 28th September 2020 (https://osf.io/7vqah/). First, we hypothesised that RWA would positively predict rule following in the Rule Following Task. Second, we hypothesised that RWA would positively predict social information use in the BEAST.

#### Statistical analysis

We pre-registered separate Bayesian multilevel models to analyse the data from both new tasks. For the Rule Following Task, we fitted a Bayesian multilevel logistic regression model with binary rule following (0 = break rule, 1 = follow rule) in each round as the outcome variable, round number and RWA as fixed-effect predictors, and random intercepts and slopes for round number grouped by participant. For the BEAST, we fitted a Bayesian multilevel beta regression model with BEAST score as the outcome variable. Beta regression is a useful technique for analysing continuous outcome variables that are bounded between 0 and 1^78^. We included RWA as the sole fixed-effect predictor, and random intercepts grouped by image type and participant. As additional exploratory analyses, we also fitted Bayesian ordinal regressions predicting policy views from rule following frequencies and BEAST scores. We used weakly-informative priors, and all models converged normally ($\hat{R}$ = 1). Model comparison was implemented using exact K-fold cross-validation^79^.

All analyses were conducted in R Version 4.0.2^63^. We used the *brms* package for Bayesian multilevel models^80^. Figures were created with the *ggplot2*^66^ and *cowplot*^67^ packages, and the manuscript was reproducibly generated with the *drake*^68^ and *papaja*^69^ packages. Code to reproduce all analyses can be found on the Open Science Framework: https://osf.io/dwx8g/^55^.

#### Ethics statement

For Study 2, an ethics amendment was granted by the University of Auckland Human Participants Ethics Committee (ref: 021666). The study was performed in accordance with all the relevant guidelines and regulations. Informed consent was obtained from all participants prior to the study.

### Results

**Figure 4:**
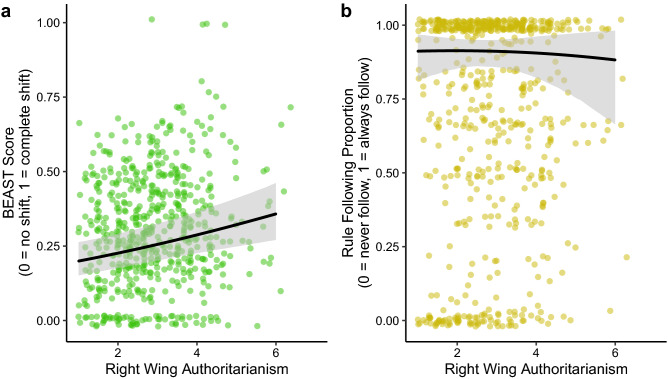
Right Wing Authoritarianism positively predicts social information use in the BEAST (**a**) but does not predict rule following in the Rule Following Task (**b**). Lines are mean posterior regression lines from Bayesian multilevel models without demographic covariates, shaded areas are 95% credible intervals.

We found that rule following was bimodally distributed, with most participants either always following the rule or always breaking it (Supplementary Fig. S9). Contrary to our first pre-registered hypothesis, we found that RWA did not predict rule following. The Spearman’s rank correlation between RWA and the number of rounds in which participants followed the rule was 0.02 (*p* = 0.673). Similarly, in a Bayesian multilevel logistic regression analysis predicting the probability of following the rule, including RWA as a fixed-effect predictor reduced model fit compared to the null model (difference in expected log pointwise predictive density = $-28.08$, *SE* = 13.75) and the 95% credible interval for the log-odds RWA slope crossed zero (*b* = $-0.03$, 95% CI [$-0.50$, 0.44]; Fig. 4b). This result held when additionally controlling for all demographics (Supplementary Table S15). Interestingly, these additional models revealed that SDO negatively predicted the probability of rule following (*b* = $-0.60$, 95% CI [$-1.15$, $-0.07$]), suggesting that high SDO participants were more likely to break the rule for personal gain. In further exploratory analyses, we found that the frequency of rule following predicted socially *progressive* acceptance of homosexuality in society (*b* = $-0.40$, 95% CI [$-0.81$, $-0.01$]) but did not predict any other social policy views.

Replicating previous work^44^, we found that BEAST scores were skewed towards zero, with most participants choosing to stick with their initial estimate rather than shift their estimate towards the social information (Supplementary Fig. S10). In line with our second pre-registered hypothesis, we found that RWA positively predicted social information use in the BEAST. In a Bayesian multilevel beta regression analysis predicting BEAST scores (0 = no shift in estimate, 1 = complete shift in estimate to social information), including RWA as a fixed-effect predictor improved model fit over the null model (difference in expected log pointwise predictive density = 26.85, *SE* = 18.38) and the 95% credible interval for the RWA slope was greater than zero (*b* = 0.16, 95% CI [0.08, 0.24]; Fig. 4a). This result held when additionally controlling for SDO, age, gender, ethnicity, education, socio-economic status, and local deprivation, individually and together in a full model (Supplementary Table S16). In contrast, SDO did not predict social information use in the BEAST (*b* = 0.05, 95% CI [$-0.04$, 0.15]).

**Figure 5:**
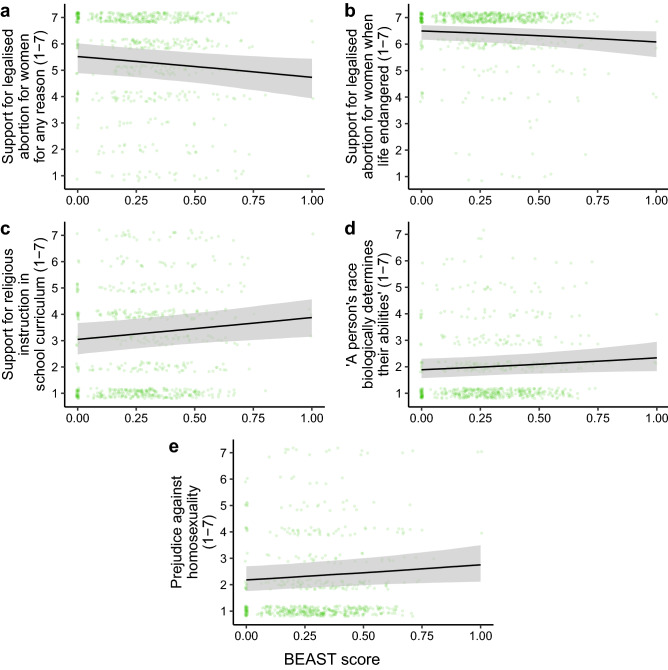
BEAST scores predict a host of socially conservative views. For every Likert scale, 7 indicates increased support for the policy or agreement with the statement, whereas 1 indicates reduced support for the policy or disagreement with the statement. Regression lines and 95% credible interval shaded areas are predictions from Bayesian ordinal models including demographic covariates.

BEAST scores also reliably predicted socially conservative views across a wide range of political issues (Fig. 5; Supplementary Tables S17–S23). Controlling for demographics, exploratory Bayesian ordinal regressions revealed that mean BEAST scores (averaged over all five rounds) positively predicted support for religious instruction in schools (*b* = 0.75, 95% CI [0.12, 1.36]), agreement with the essentialist belief that “a person’s race biologically determines their abilities” (*b* = 0.62, 95% CI [$-0.04$, 1.28]), and disagreement with homosexuality being accepted by society (*b* = 0.64, 95% CI [0.01, 1.29]). BEAST scores also negatively predicted support for abortion, both for any reason (*b* = $-0.76$, 95% CI [$-1.41$, $-0.12$]) and specifically when the woman’s life is in danger (*b* = − 0.76, 95% CI [$-1.46$, $-0.05$]). The only social issues not predicted by BEAST scores were support for same-sex marriage (*b* = $-0.34$, 95% CI [$-1.00$, 0.30]) and euthanasia (*b* = −0.53, 95% CI [−1.18, 0.11]), although these effects were in the expected direction. In contrast, BEAST scores did not predict any of the economic views predicted by the cooperative phenotype in Study 1.

### Discussion

We found that RWA, but not SDO, predicted increased conformity as measured by social information use in the BEAST. This builds on previous work with this task showing that social information use is associated with collectivist values^44^. Furthermore, we found that individual differences in social information use predicted socially conservative views across a wide range of political issues, such as abortion, homosexuality, and religious instruction in schools, but did not predict economic views. By relating RWA and social policy views to conformist preferences, these findings provide further support for the dual evolutionary framework of political ideology.

As with our findings with the norm-enforcing punishment games, we found no relationship between RWA and rule following, but we did find a relationship with SDO. One explanation for this is that, while intended to track willingness to follow a rule, the Rule Following Task also reflects elements of competitiveness, selfishness, and striving for personal gain: by breaking the rules, individuals can earn more money for themselves. The Rule Following Task also exogenously imposes a rule, but from the instructions alone it is unclear who has imposed the rule or for what reason. We might have found a different pattern of results if participants had been told that the rule had been imposed by another participant or by the consensus of their in-group.

## General discussion

Across two pre-registered studies, we found that behaviour in incentivised tasks was related to the two dimensions of political ideology and a wide variety of policy views. Participants who cooperated more in economic games were lower in SDO and expressed economically progressive policy views. Cooperation was unrelated to RWA or social views. Likewise, participants who conformed more to social information were higher in RWA and expressed socially conservative policy views. Social information use was unrelated to SDO or economic views. However, we also found that punishment and rule following behaviour did not show the predicted relationship with RWA or socially conservative policy views, and instead covaried with SDO. Taken together, these results provide qualified support for and refine the general claim made by the dual evolutionary framework that the two dimensions of political ideology reflect phenotypic variation in preferences for cooperation and conformity.

We found that SDO and economic policy views were related to a cooperative phenotype latent variable estimated from multiple cooperation games. SDO was also related to most individual cooperative decisions, including decisions reflecting trust, altruism, coordination, and collective action. Recent work has characterised lower SDO and economically progressive views as specifically related to a reduced willingness to exploit others for personal gain^39^ and greater interpersonal trust^81^. Our exploratory analyses revealed that SDO is indeed related to exploitative behaviour, including returning less in the Trust Game, but it is also related to less cooperative behaviour in games that do not involve a conflict of interest and so do not allow for exploitation of others, such as not coordinating to mutual benefit in the Stag Hunt Game. Together, these findings support the prediction from the dual evolutionary framework that low SDO and support for economically progressive policies reflect a *general* preference for cooperation that shapes people’s views across a myriad of different social dilemmas that emerge from human group living, such as welfare benefit programmes, taxation systems, and collective responses to climate change.

We found that RWA and social policy views were related to conformity in the form of social information use. In contrast, RWA and social policy views were unrelated to norm-enforcing punishment or general rule following. Taking these results at face value, this suggests that, contra our pre-registered predictions, authoritarian and socially conservative views might be better characterised by informational conformity than normative conformity^82^. Informational conformity functions to adapt individual behaviour to local conditions by learning from peer behaviour, whereas normative conformity functions to minimise peer disapproval via enforcement of and adherence to social norms^83^. A link between RWA and informational conformity fits with research showing that conservatives are more likely to share similar judgements and attitudes with like-minded people and exaggerate informational consensus within groups^84^, as well as prior work linking social information use to collectivist values^85^. The conclusion that normative conformity does not differ across the political spectrum, if warranted, would also align with research showing that conservatives and liberals are equally likely to enforce their group’s social norms and exhibit intolerance towards out-groups with different norms^86^. If this characterisation of authoritarian and socially conservative ideology is correct, we will need to refine the dual evolutionary framework of political ideology to place less emphasis on group-wide social norms and more emphasis on in-group informational conformity and social information use.

However, it is also possible that our norm-enforcing punishment and rule following tasks were poor measures of a general group conformist preference. As noted above, these tasks contain competitive and selfish elements that may explain their correlations with SDO rather than RWA. The tasks did not explicitly measure social norms^87^ or allow players to interact over multiple rounds, limiting the potential for the emergence and enforcement of normative expectations. The tasks were also abstract and context-neutral: they did not specify who the players in the interaction were or contain any differentiation between in-groups and out-groups. This avoids conflating participants’ general predisposition to conform with their identification with a particular group, but may fail to activate conformist preferences that depend on specific group contexts, such as socially conservative group identities^12,51^. Such an explanation would imply that authoritarians and social conservatives conform to social information in all contexts, but will only enforce and adhere to norms in specific group contexts. Future research could adapt the methods used here to further refine our understanding of the behavioural preferences underlying authoritarianism and social conservatism. In such work, it would be informative to relate preferences to specific ideological subdimensions, such as authoritarian aggression or authoritarian submission^88^, in order to better map the behavioural constellation of the conformist dimension of political ideology.

There are several important limitations of this work that should guide future research. First, while we collected data from a large, diverse adult sample, it remains unclear to what extent our findings apply outside of New Zealand, both in other developed countries and in non-WEIRD^89^ and small-scale societies. Future work should replicate these results in other populations around the world, including using adapted behavioural measures to predict locally-relevant political attitudes in small-scale societies. Second, the effect sizes in both studies were relatively small. However, smaller effect sizes are to be expected from relationships between behaviour and self-reported attitudes^90^ and do not preclude these relationships from being consequential in the long run^91^. Third, it is possible that the associations between behaviour and political views could have simply arisen from people mapping their prior political beliefs onto the tasks. We believe this is unlikely, since the tasks were abstract and contained no political framing. Future work examining longitudinal data could expand on our cross-sectional study to formally test the directions of causality between general preferences and political attitudes.

In sum, we have shown clear differences in social preferences across the two dimensions of political ideology. We found that a general cooperative preference covaried with SDO and economic policy views, and informational conformity covaried with RWA and social policy views. These psychological differences were largely consistent with predictions of the dual evolutionary framework of political ideology^5^. The predictions not supported by the data suggest that the relationship between informational conformity and RWA and social policy views may not extend to conformity in the form of norm-enforcement and rule following, although demonstrating this conclusively will require further research using alternative behavioural measures. Taken together, our findings reveal the very different social preferences underlying both ideological dimensions, highlighting the importance of work that moves beyond unidimensional scales to elucidate the dual evolutionary foundations of political ideology.

## Supplementary Information

Supplementary Information.

## Data Availability

A copy of the anonymous data reported in each New Zealand Attitudes and Values Study publication is available from Professor Chris Sibley (email:c.sibley@auckland.ac.nz) upon request from appropriately qualified researchers. Such data will be provided with the explicit understanding that it is used solely for the purposes of replicating or otherwise checking the validity of analyses reported in scientific papers analysing New Zealand Attitudes and Values Study data.

## Code availability

Python code for the incentivised behavioural tasks and R code for the statistical analyses, figures, and manuscript generation are publicly available at https://osf.io/dwx8g/.
