# Supplementary material for: Cooperative and conformist behavioural preferences predict the dual dimensions of political ideology
Source: Sci Rep. 2023 Mar 25;13:4886. doi: 10.1038/s41598-023-31721-6 (PMC10039865; doi:10.1038/s41598-023-31721-6)
Supplement: Supplementary file 1 — Supplementary Information. [file 41598_2023_31721_MOESM1_ESM.pdf]

Supplementary Materials

Supplementary Methods

Power analysis. The minimum size of our sample was determined by conducting a power analysis on data from previous work[47]. We used the smallest significant correlation between economic game play and real-world cooperation in that study as the effect size (r = 0.15). G*Power[92] suggested a sample of 571 participants to detect this correlation effect size with statistical power of 0.95. We aimed to sample 1000 participants, substantially above this amount, and eventually report data from 991 participants.

Sample frame in Study 1. From the New Zealand Attitudes and Values Study, we included participants in our sample frame who: had completed Wave 4 of the study (n = 12,189); had also completed Wave 9 and/or Wave 10 (n = 8,095); had not subsequently withdrawn from the study at the time of sampling (n = 7,833); had consistently indicated at Wave 9 and 10 that they would be willing to participate in an additional online study (n = 4,181); had a valid email address (n = 4,040); were living in New Zealand (n = 3,955); were younger than 70 at the time of sampling (n = 3,374); and had a valid cell or landline number (n = 3,345). Of these 3,345 participants, we attempted to contact 3,063 about an additional study involving "economic decision-making in groups", and successfully contacted 2,731 about the study.

Additional information about study sessions in Studies 1 and 2. In Study 1, participants expressed interest in an initial phone call and were then emailed further information about the study. In Study 2, they were just sent an initial email. In both studies, the email asked them to complete a Qualtrics survey to specify their availability the following week. This survey removed any ineligible participants who (1) did not have adequate access to the Internet, (2) did not have a quiet place to participate in the study, and (3) did not have a New Zealand bank account (for payment purposes).

At the beginning of the study, participants first completed a consent form, in which they were informed of their confidentiality, right to withdraw, payment, and ethical approval. If they agreed to the consent form, participants read information about the economic games and then completed the games in a random order. After being randomly matched at the end of the session, participants saw a payoff screen which summarised the results of each individual game and how much they had earned from the study. Name and bank account details were collected at the end of the study. For security purposes, this information was encrypted while stored online and later decrypted on a local computer for payment.

Participants had a 55 minute limit to complete the games. If participants took longer than 55 minutes, the software skipped them ahead to the waiting lobby. Timeouts were still paid the $20 show-up fee, but no bonus payment.

Uneven session sizes in Studies 1 and 2. In Studies 1 and 2, session sizes were often not strict multiples of four necessary for real-time random matching, either due to unforeseen dropouts or uneven sign-ups. To remedy this, "simulated players" were used to round up uneven session sizes. These simulated players chose responses in the games based on the median responses from previous work[47]. Participants were made aware that they may be matched with simulated players, but only on the payoff screen *after* all game decisions had been made: "In the rare event that we could not find a participant to match you with, we have instead matched you with average decisions based on previous research."

Supplementary Results

Confirmatory factor analyses in Study 1. As with our principal components analysis, we initially restricted our confirmatory factor model to include only the games used in previous work[47]. Controlling for game comprehension, we loaded the Dictator Game, Trust Game (Give), Trust Game (Return), and Public Goods Game onto the cooperation latent variable, and loaded the Ultimatum Game (Minimum Acceptable Offer), Third-Party Punishment Game (Punish), and Second-Party Punishment Game (Punish Defector) onto the punishment latent variable. According to established fit statistic cutoffs[93,94], this model fitted the data well, with a Root Mean Square Error of Approximation (RMSEA) value of 0.05, a Standardised Root Mean Square Residual (SRMR) value of 0.04, and a Comparative Fit Index (CFI) value of 0.93. All indicators had significantly positive loadings, and the two latent variables were significantly negatively correlated with one another (r = -0.17, p = .004; Supplementary Figure S11). Model fit improved further when additionally loading the Stag Hunt Game onto the cooperation latent variable and loading the Stag Hunt Game with Punishment (Punish Defector) onto the punishment latent variable (RMSEA = 0.04; SRMR = 0.04; CFI = 0.95; Supplementary Figure S12).

Confirmatory factor analysis with method factor in Study 1. It is possible that method factors could be influencing the economic game data and generating a two-dimensional factor structure. For example, participants could simply be responding positively to all the games, regardless of their content. To test for the influence of method effects, we also included an additional method factor in the confirmatory factor model. This method factor had loadings from all game decisions, which were all constrained to 1 (i.e., a random intercept), and covariances with the cooperation and punishment factors constrained to 0. Factor loadings were relatively unchanged in this model, with all game decisions loading positively and significantly onto their respective factors (Supplementary

Figure S13). The model continued to fit the data well (RMSEA = 0.03; SRMR = 0.04; CFI = 0.98). These results suggest that method factors are not driving the two-dimensional structure of gameplay.

Structural equation model with method factor in Study 1. We also included a method factor, as described above, in our structural equation model to check the robustness of the relationships between gameplay and political ideology to method effects. In this model, we found that SDO continued to significantly negatively predict the cooperation latent variable with a small-to-medium effect size (standardised β = -0.25, unstandardised b = -0.04, 95% confidence interval [-0.05, -0.02], $p < .001$). SDO also continued to significantly positively predict the punishment latent variable with a small effect size (β = 0.14, b = 0.005, 95% CI [0.002, 0.008], p = .004). As in our initial model, we found no significant relationship between RWA and the punishment latent variable (β = -0.01, b = 0.000, 95% CI [-0.003, 0.002], p = .823) or between RWA and the cooperation latent variable (β = -0.02, b = 0.00, 95% CI [-0.01, 0.01], p = .580). These results show that method factors cannot explain the observed relationships between gameplay and political ideology.

Confirmatory factor analyses and structural equation models with robust estimator. We refitted our main models using a robust estimator (weighted least square mean and variance adjusted estimator) to ensure that our inferences are not biased due to non-normal and ordinal response distributions.

When fitted with this robust estimator, our first confirmatory factor model fitted the data well (RMSEA = 0.05; SRMR = 0.04; CFI = 0.93). This model loaded cooperative behaviour in the Dictator Game, the Trust Game, and the Public Goods Game onto the "cooperation" latent variable, and punishment of defectors in the Ultimatum Game, Third-Party Punishment Game, and Second-Party Punishment Game onto the "punishment" latent variable. As in our main model, the two latent variables were significantly negatively correlated with one another (r = -0.17, p = .027). Model fit

improved further when additionally loading the Stag Hunt Game onto the cooperation latent variable and loading the Stag Hunt Game with Punishment (Punish Defector) onto the punishment latent variable (RMSEA = 0.04; SRMR = 0.04; CFI = 0.95).

We additionally fitted our main structural equation model, including SDO and RWA as predictors of the two latent variables, with the robust estimator. Replicating the result of our main model, we found that SDO negatively predicted the cooperation latent variable (standardised β = -0.24, unstandardised b = -0.04, 95% confidence interval [-0.05, -0.02], $p < .001$) and positively predicted the punishment latent variable (standardised β = 0.13, unstandardised b = 0.01, 95% confidence interval [0.00, 0.01], $p = .003$). By contrast, RWA was unrelated to both the cooperation latent variable (standardised β = -0.02, unstandardised b = 0.00, 95% confidence interval [-0.01, 0.01], $p = .582$) and the punishment latent variable (standardised β = -0.01, unstandardised b = 0.000, 95% confidence interval [-0.003, 0.002], $p = .882$).

Supplementary Figures

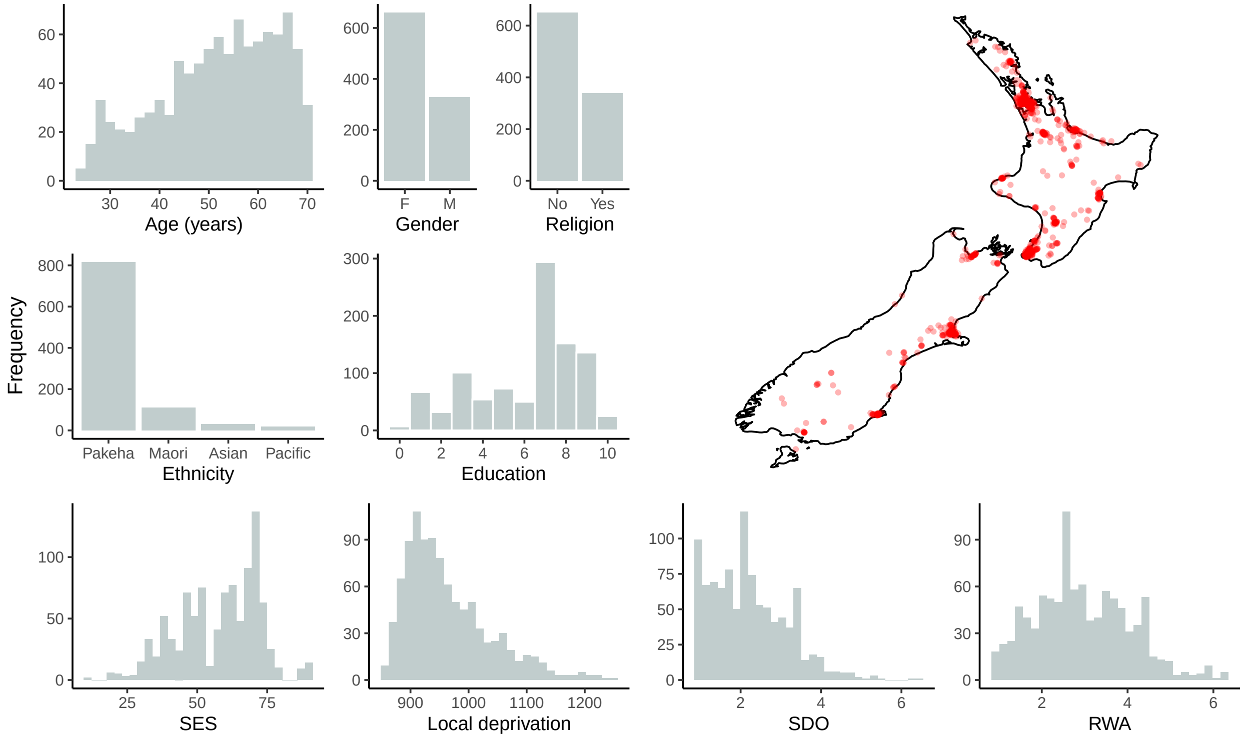

Figure S1. Final sample characteristics (n = 991). Education is measured by NZREG, an ordinal scale measuring level of educational attainment (range = 0-10). Socio-economic status (SES) is measured by NZSEI, an ordinal rank measure based on occupational categories (range = 10-90). Local deprivation is measured by NZDEP, an ordinal rank measure of local deprivation for each meshblock in New Zealand (range = 840-1550). Social Dominance Orientation (SDO) and Right Wing Authoritarianism (RWA) are mean scores from six Likert scales (range = 1-7). The map of New Zealand shows meshblock locations for participants.

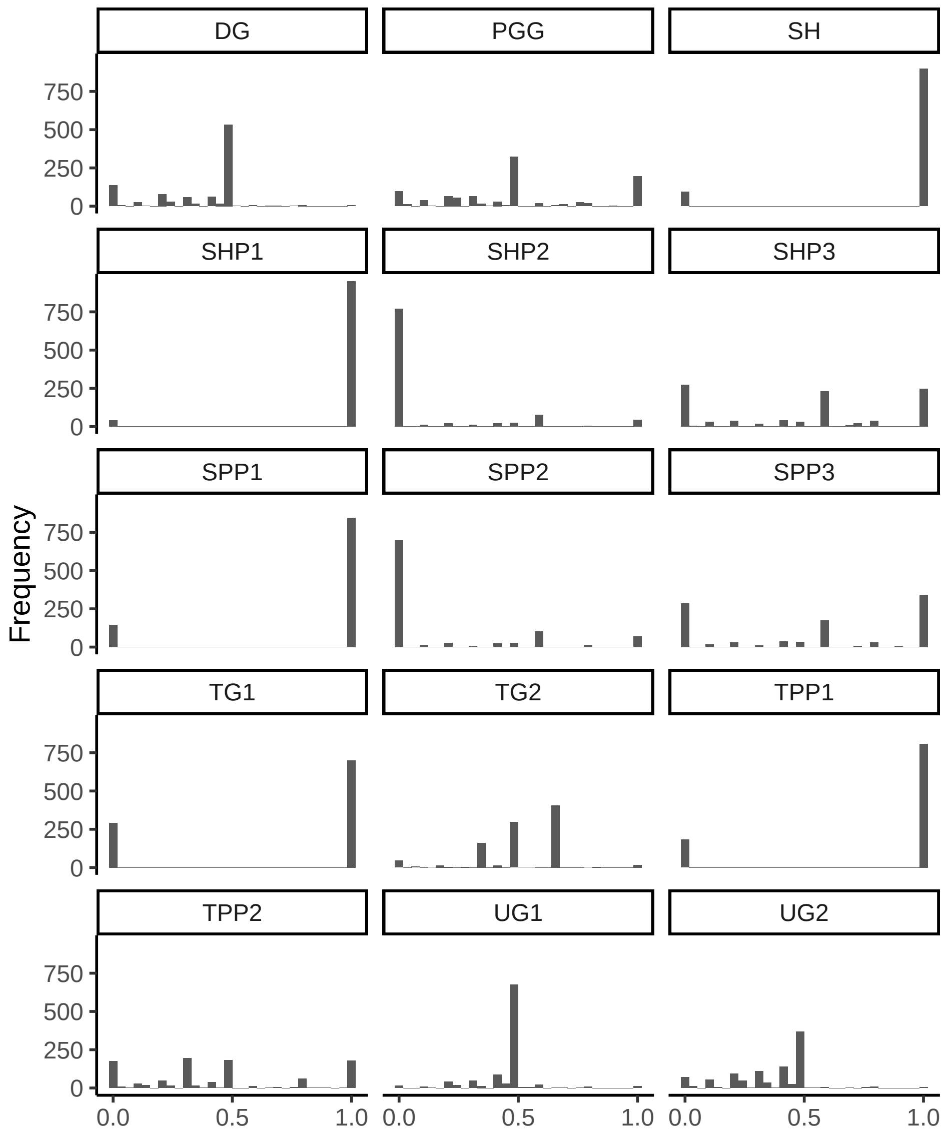

Figure S2. Histograms showing distributions of decisions in the economic games in Study 1. Each game decision was normalised to vary between 0 and 1. Counts on the y-axis represent numbers of participants. DG = Dictator Game, PGG = Public Goods Game, SH = Stag Hunt Game, SHP1 = Stag Hunt Game with Punishment (Coordinate), SHP2 = Stag Hunt Game with Punishment (Punish Coordinator), SHP3 = Stag Hunt Game with Punishment (Punish Defector), SPP1 = Second-Party Punishment Game (Cooperate), SPP2 = Second-Party Punishment Game (Punish Cooperator), SPP3 = Second-Party Punishment Game (Punish Defector), TG1 = Trust Game (Give), TG2 = Trust Game (Return), TPP1 = Third-Party Punishment Game (Steal), TPP2 = Third-Party Punishment Game (Punish), UG1 = Ultimatum Game (Offer), UG2 = Ultimatum Game (Minimum Acceptable Offer).

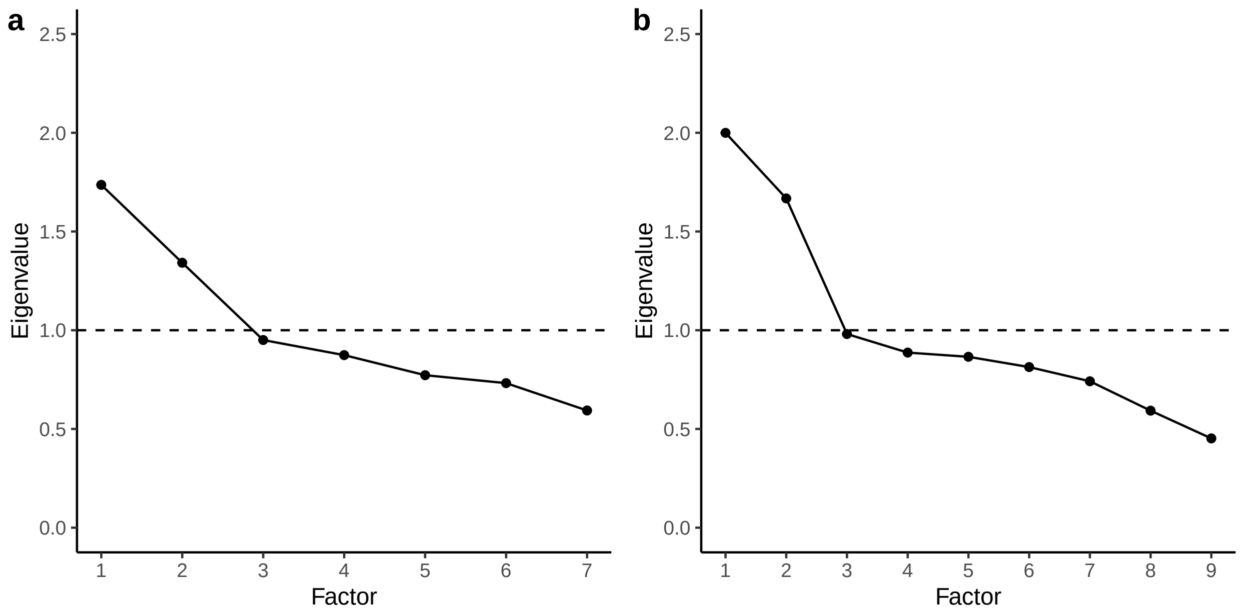

Figure S3. *Scree plots for (a) the principal components analysis in Study 1 with only the game decisions from previous work and (b) the extended principal components analysis in Study 1 including coordination games.* Principal components analyses initially contained as many factors as there were variables. Both scree plots show that only the first two factors have eigenvalues above 1, indicating a two-factor solution.

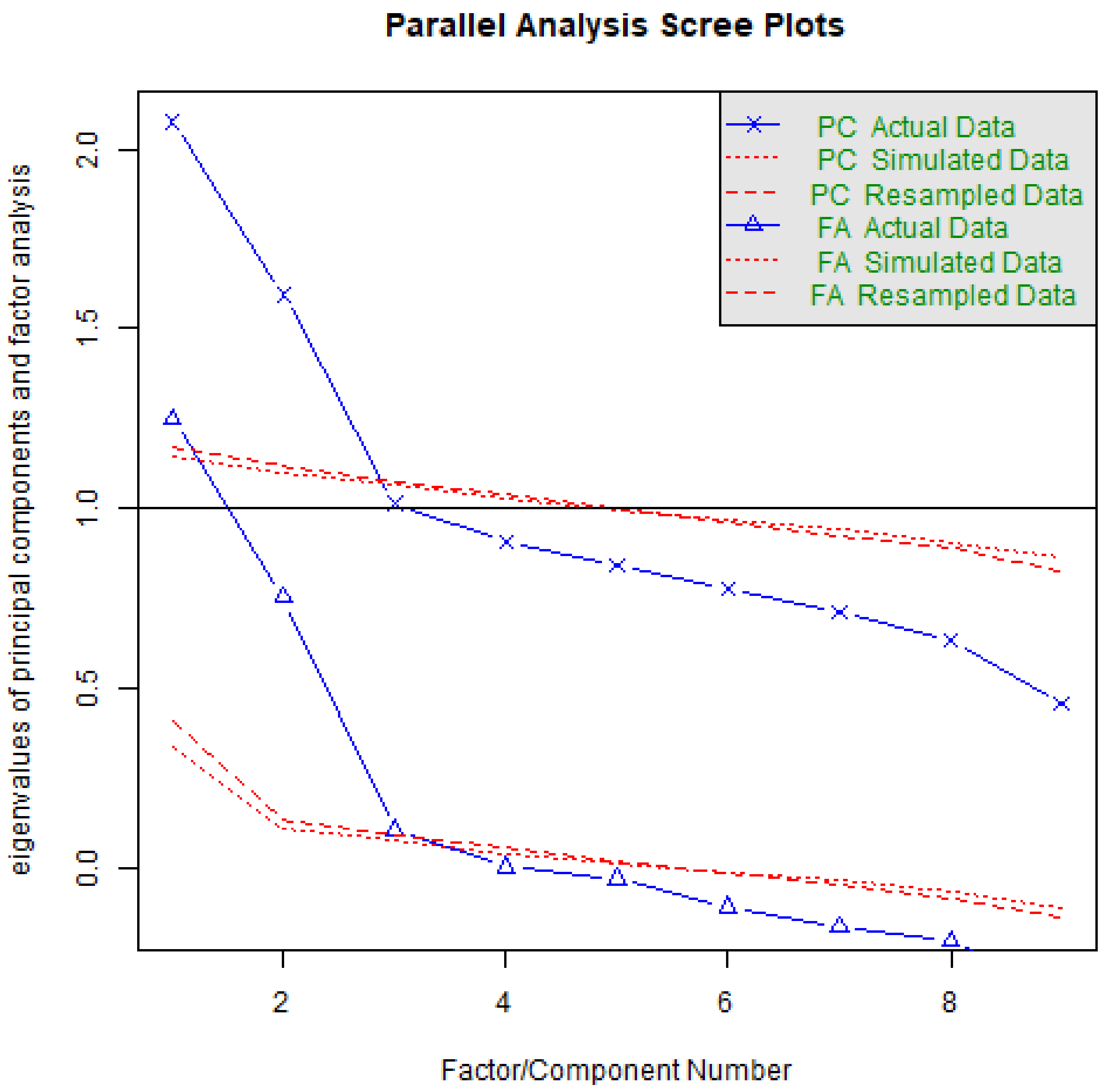

Figure S4. Scree plot from exploratory parallel analysis of economic game decisions. The parallel analysis supported a two-factor solution.

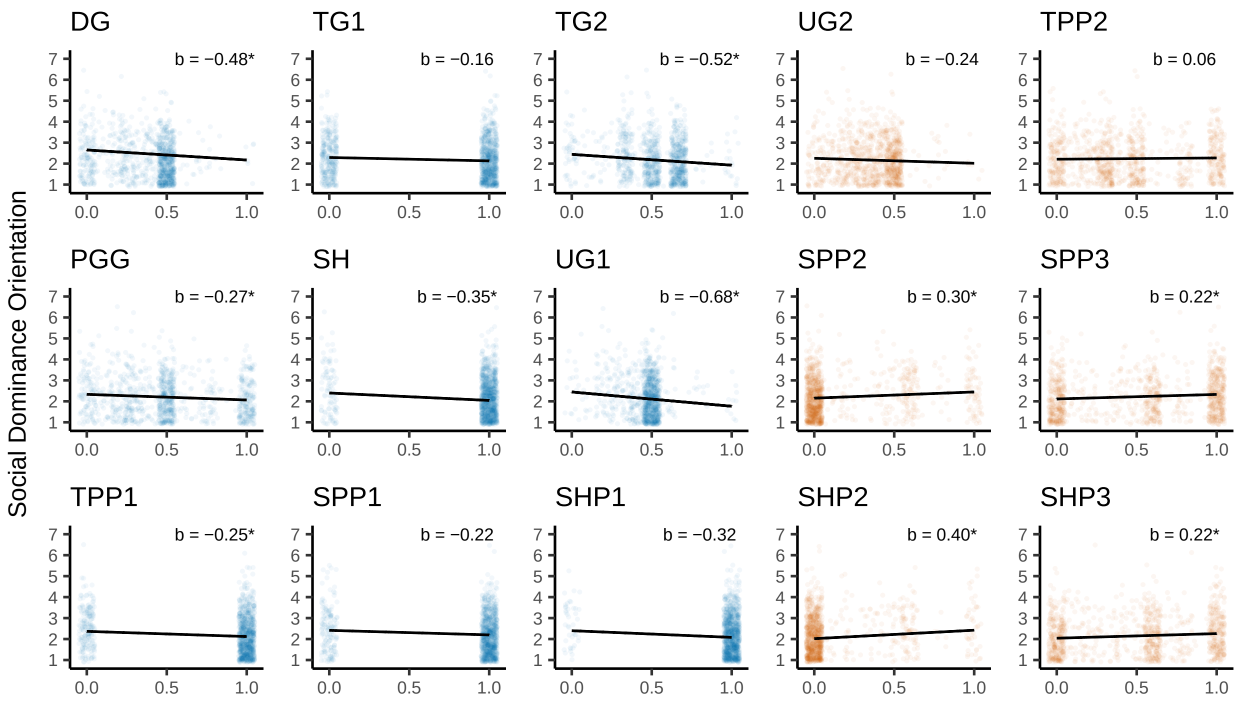

Figure S5. Individual game decisions predicting Social Dominance Orientation in Study 1. Lines are predictions from linear regressions, controlling for Right Wing Authoritarianism, game comprehension, and demographics. Multiple comparisons are accounted for by using a Bonferonni-corrected alpha level. Blue plots are cooperation decisions, orange plots are punishment decisions. Numbers are unstandardised coefficients. $^*p < .003$. DG = Dictator Game, TG1 = Trust Game (Give), TG2 = Trust Game (Return), PGG = Public Goods Game, SH = Stag Hunt Game, UG1 = Ultimatum Game (Offer), TPP1 = Third-Party Punishment Game (Steal), SPP1 = Second-Party Punishment Game (Cooperate), SHP1 = Stag Hunt Game with Punishment (Coordinate), UG2 = Ultimatum Game (Minimum Acceptable Offer), TPP2 = Third-Party Punishment Game (Punish), SPP2 = Second-Party Punishment Game (Punish Cooperator), SPP3 = Second-Party Punishment Game (Punish Defector), SHP2 = Stag Hunt Game with Punishment (Punish Coordinator), SHP3 = Stag Hunt Game with Punishment (Punish Defector).

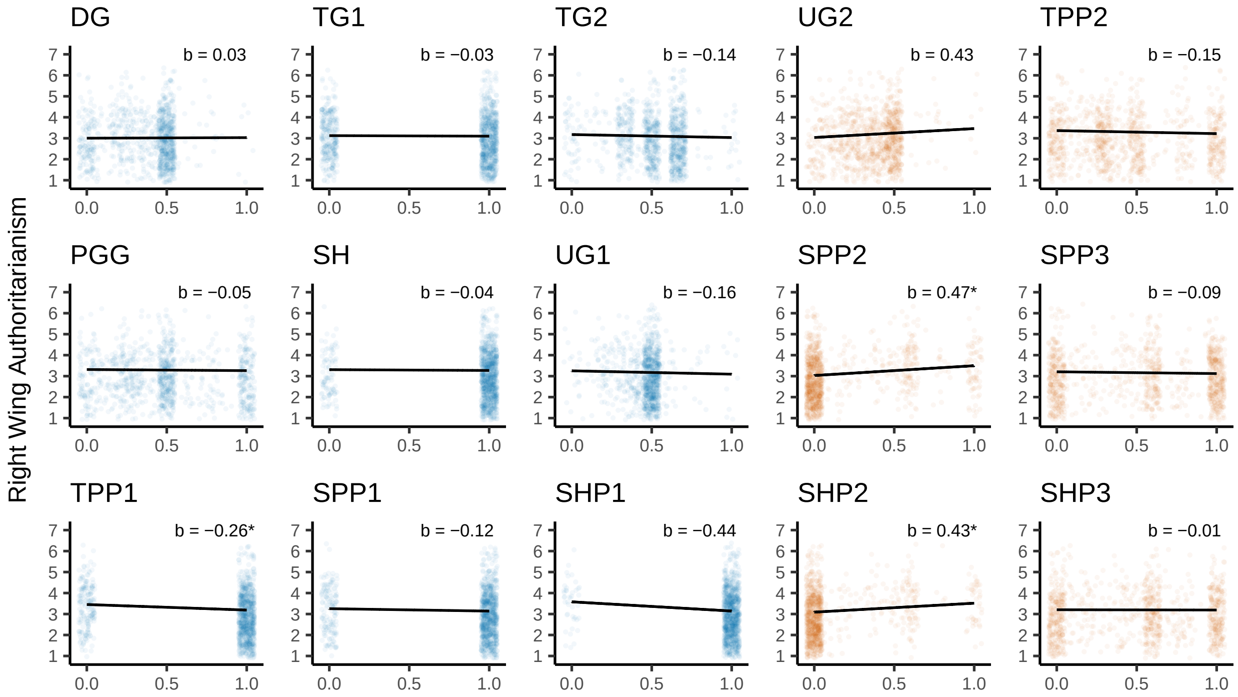

Figure S6. Individual game decisions predicting Right Wing Authoritarianism in Study 1. Lines are predictions from linear regressions, controlling for Social Dominance Orientation, game comprehension, and demographics. Multiple comparisons are accounted for by using a Bonferonni-corrected alpha level. Blue plots are cooperation decisions, orange plots are punishment decisions. Numbers are unstandardised coefficients. $^{*}p < .003$. DG = Dictator Game, TG1 = Trust Game (Give), TG2 = Trust Game (Return), PGG = Public Goods Game, SH = Stag Hunt Game, UG1 = Ultimatum Game (Offer), TPP1 = Third-Party Punishment Game (Steal), SPP1 = Second-Party Punishment Game (Cooperate), SHP1 = Stag Hunt Game with Punishment (Coordinate), UG2 = Ultimatum Game (Minimum Acceptable Offer), TPP2 = Third-Party Punishment Game (Punish), SPP2 = Second-Party Punishment Game (Punish Cooperator), SPP3 = Second-Party Punishment Game (Punish Defector), SHP2 = Stag Hunt Game with Punishment (Punish Coordinator), SHP3 = Stag Hunt Game with Punishment (Punish Defector).

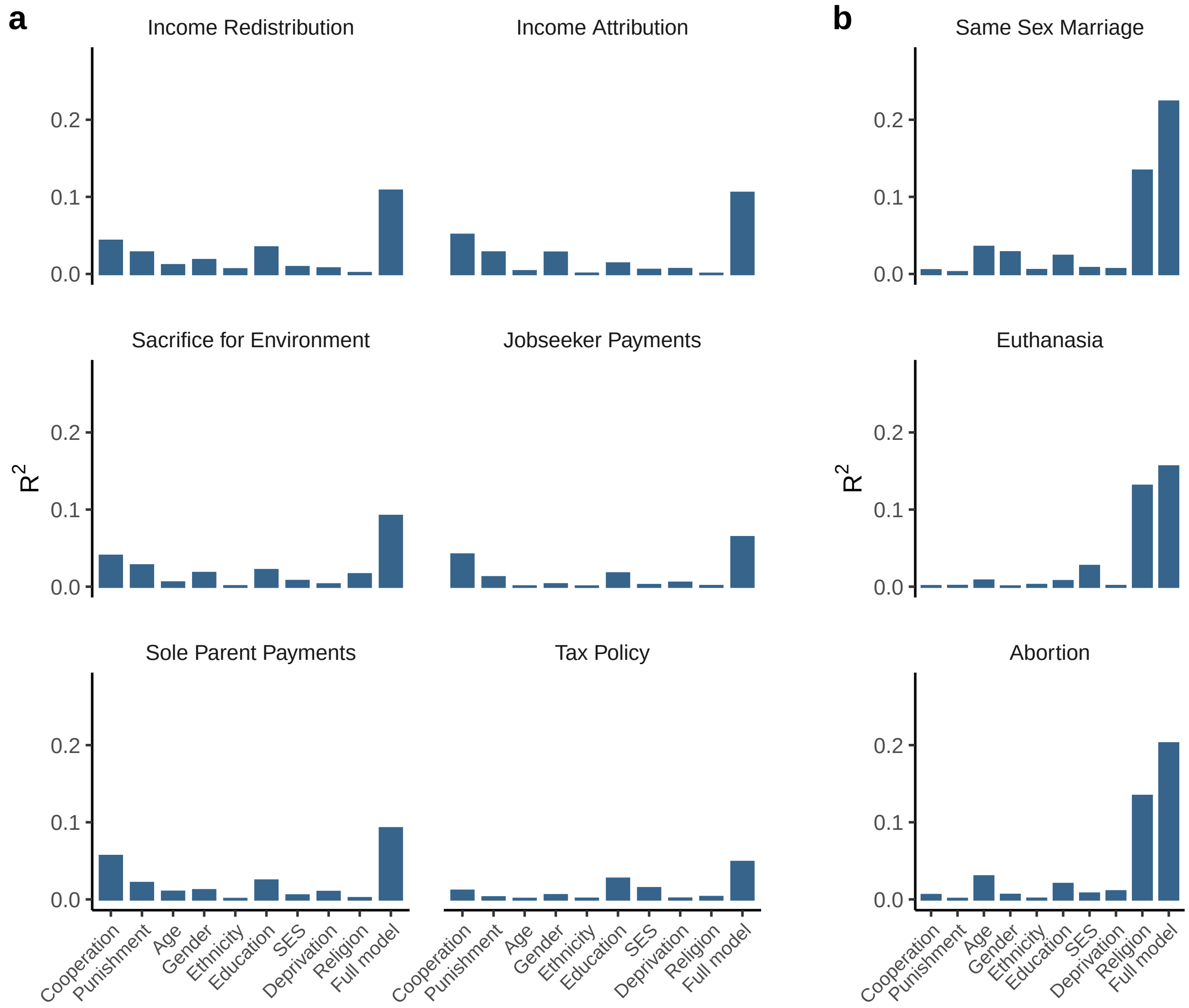

Figure S7. Bar plots for (a) economic and (b) social policy views showing the proportion of variance explained (R^2) by the cooperation latent variable, the punishment latent variable, and various demographic controls individually, and their combination in a full model. In Study 1, the cooperation latent variable explains a comparable proportion of variance in policy views to other demographics.

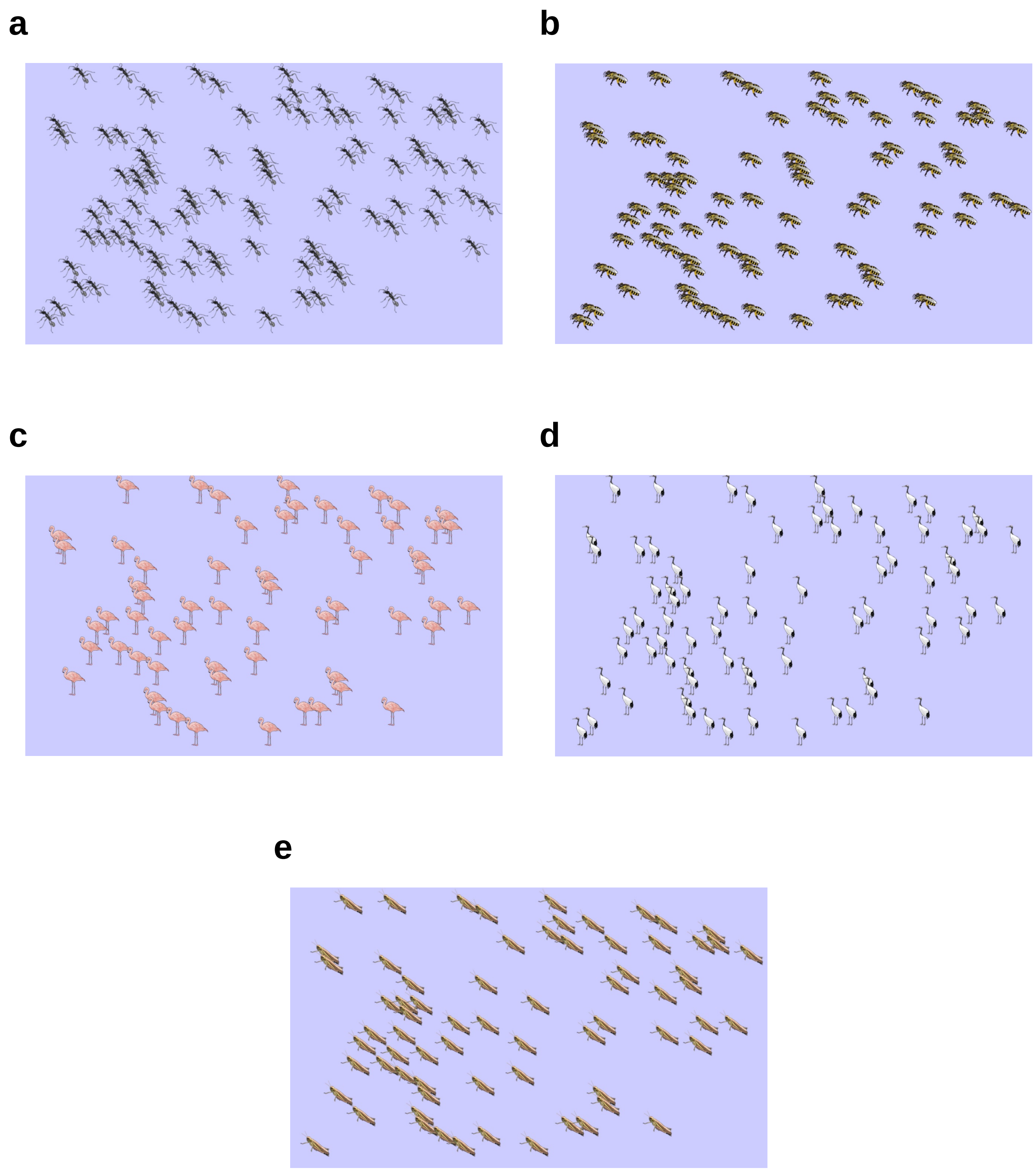

Figure S8. *Images used in the BEAST task in rounds 1-5 (a-e).* These images were generated by adapting LIONESS code available at https://github.com/LucasMolleman/Molleman_Kurvers_vandenBos_EHB2019_BEAST

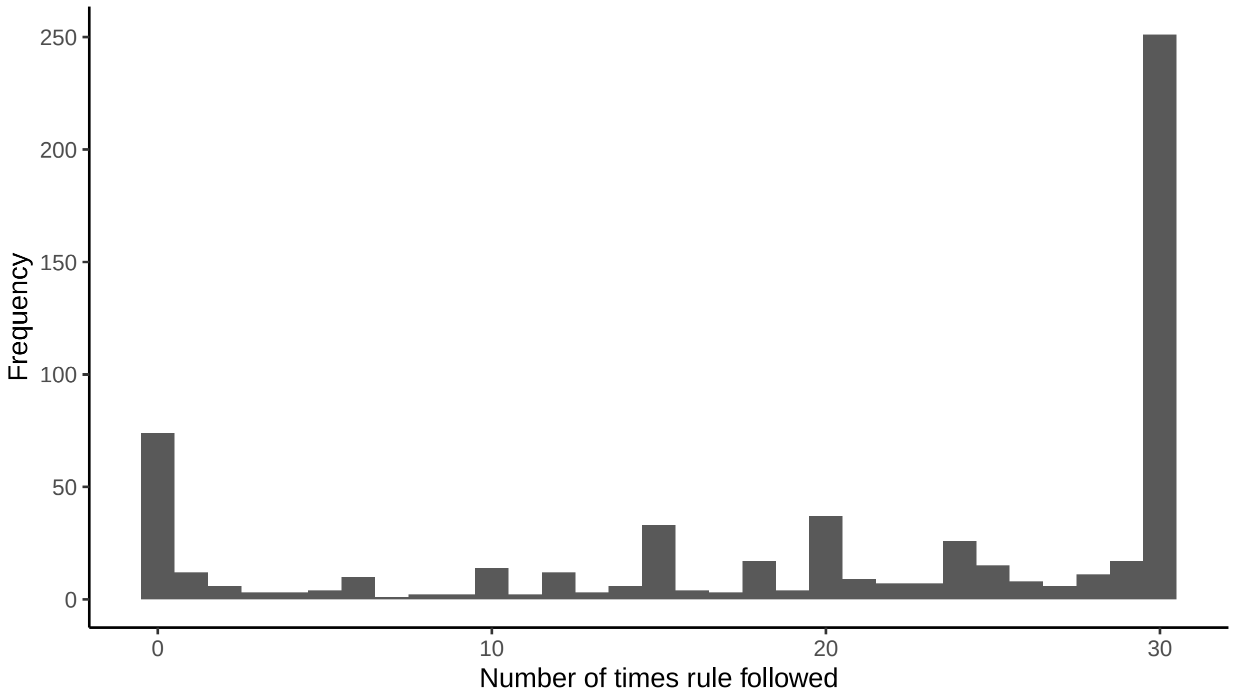

Figure S9. *Histogram of rule following decisions in the Rule Following Task in Study 2.*

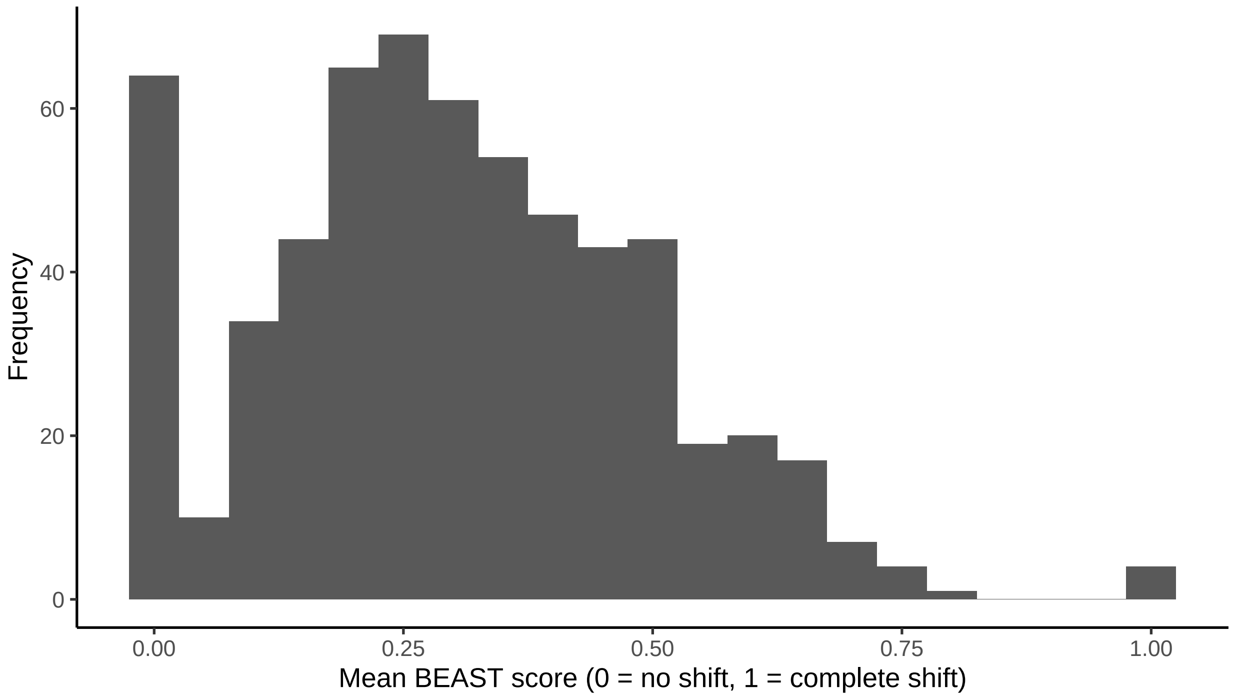

Figure S10. *Histogram of BEAST scores (averaged across all five rounds) in Study 2.*

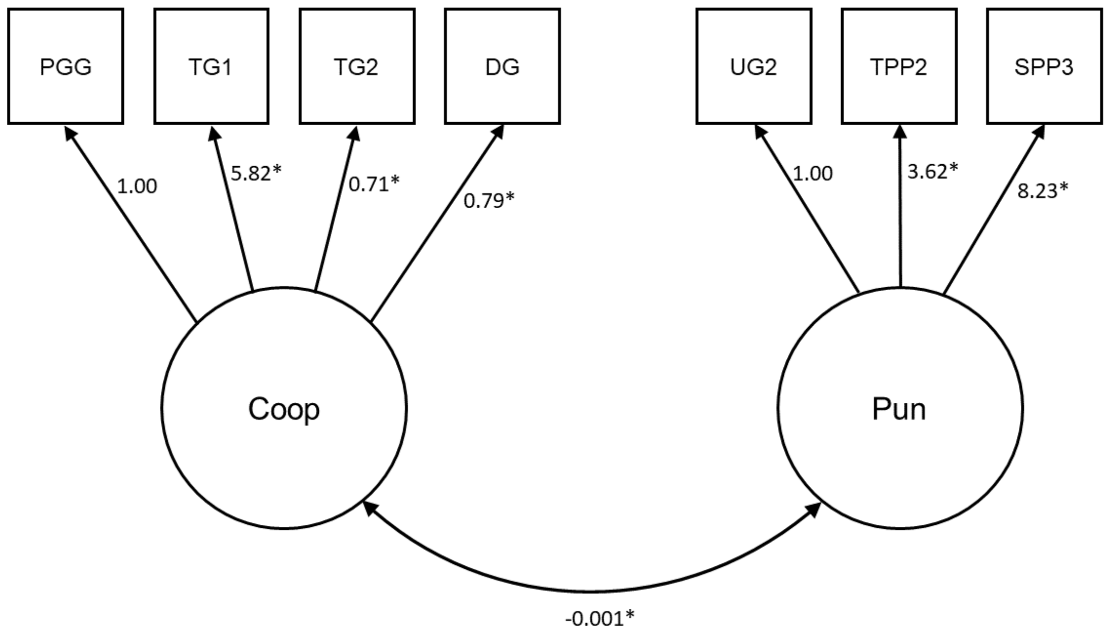

Figure S11. Confirmatory factor model in Study 1 using only the game decisions from previous work. TG1 is treated as a binary endogenous variable, and paths for PGG and UG2 are constrained to 1. Note that the regression paths controlling for game comprehension are not included in this figure. Numbers are unstandardised coefficients. $*p < .050$. PGG = Public Goods Game, TG1 = Trust Game (Give), TG2 = Trust Game (Return), DG = Dictator Game, UG2 = Ultimatum Game (Minimum Acceptable Offer), TPP2 = Third-Party Punishment Game (Punish), SPP3 = Second-Party Punishment Game (Punish Defector).

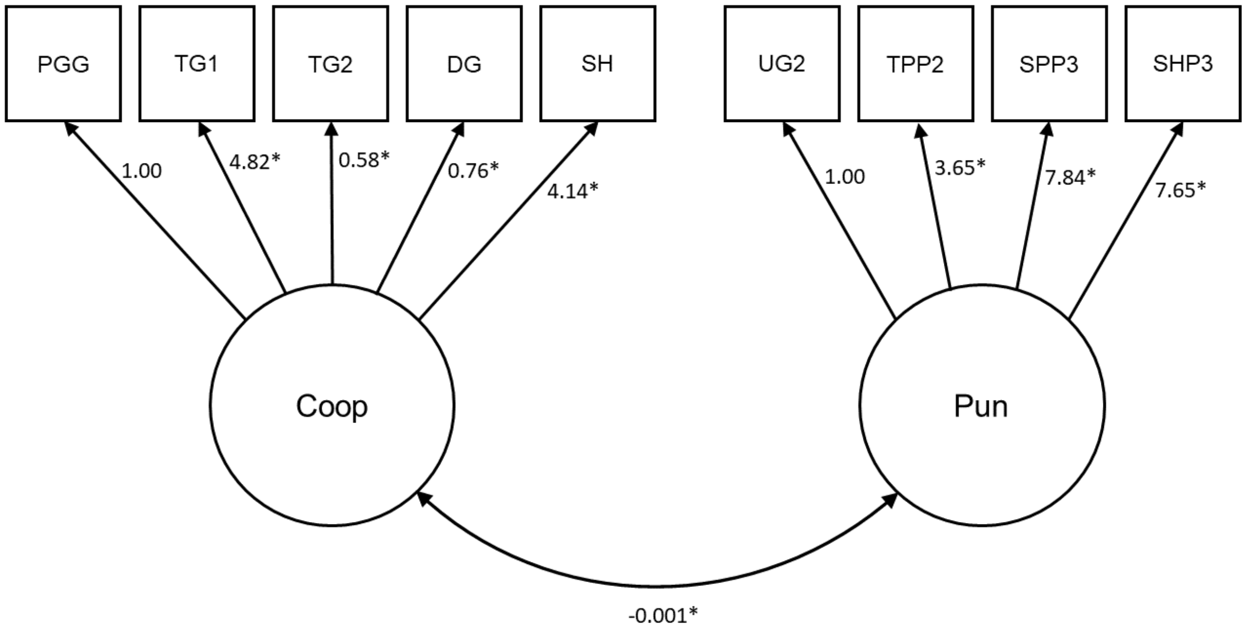

Figure S12. Confirmatory factor model in Study 1 with additional coordination games. TG1 and SH are treated as binary endogenous variables, and paths for PGG and UG2 are constrained to 1. Note that the regression paths controlling for game comprehension are not included in this figure. Numbers are unstandardised coefficients. $*p < .050$. PGG = Public Goods Game, TG1 = Trust Game (Give), TG2 = Trust Game (Return), DG = Dictator Game, SH = Stag Hunt Game, UG2 = Ultimatum Game (Minimum Acceptable Offer), TPP2 = Third-Party Punishment Game (Punish), SPP3 = Second-Party Punishment Game (Punish Defector), SHP3 = Stag Hunt Game with Punishment (Punish Defector).

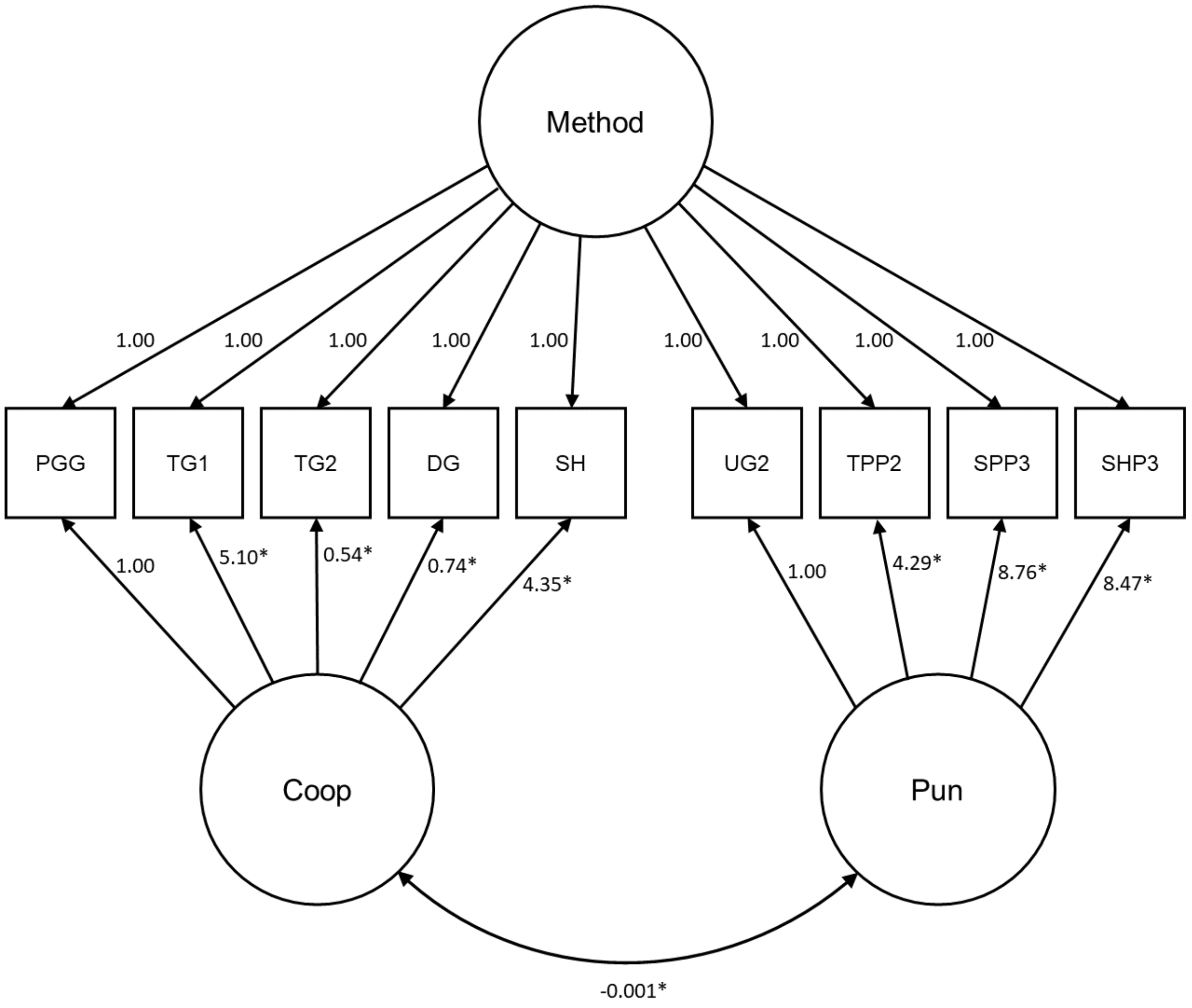

Figure S13. Confirmatory factor model in Study 1 with additional coordination games and a shared method factor. TG1 and SH are treated as binary endogenous variables, and paths for PGG and UG2 are constrained to 1. All loadings for the method factor are constrained to 1. Note that the regression paths controlling for game comprehension are not included in this figure. Numbers are unstandardised coefficients. $^*p < .050$. PGG = Public Goods Game, TG1 = Trust Game (Give), TG2 = Trust Game (Return), DG = Dictator Game, SH = Stag Hunt Game, UG2 = Ultimatum Game (Minimum Acceptable Offer), TPP2 = Third-Party Punishment Game (Punish), SPP3 = Second-Party Punishment Game (Punish Defector), SHP3 = Stag Hunt Game with Punishment (Punish Defector).

Supplementary Tables

Table S1

Factor loadings from an exploratory factor analysis of all items from the 16-item SDO scale[53] and the 30-item RWA scale[54]. The factor model was fitted to a separate validation dataset of New Zealand participants (n = 8345). The twelve items used in the New Zealand Attitudes and Values Study are emphasised in bold.

Item	Text	Factor1	Factor2
**RWA01**	**It is always better to trust the judgment of the proper authorities in government and religion than to listen to the noisy rabble rousers in our society who are trying to create doubt in people's minds.**	**0.54**	**-0.09**
**RWA02**	**It would be best for everyone if the proper authorities censored magazines so that people could not get their hands on trashy and disgusting material.**	**0.72**	**-0.25**
**RWA03**	**Our country will be destroyed someday if we do not smash the perversions eating away at our moral fibre and traditional beliefs.**	**0.56**	**0.00**
**RWA04r**	**People should pay less attention to the Bible and the other old forms of religious guidance, and instead develop their own personal standards of what is moral and immoral.**	**0.44**	**0.01**
**RWA05r**	**Atheists and others who have rebelled against the established religions are no doubt every bit as good and virtuous as those who attend church regularly.**	**0.48**	**0.10**

Table S1 continued

Item	Text	Factor1	Factor2
**RWA06r**	**Some of the best people in our country are those who are challenging our government, criticizing religion, and ignoring the "normal way things are supposed to be done."**	**0.36**	**0.05**
RWA07	Our country desperately needs a mighty leader who will do what has to be done to destroy the radical new ways and sinfulness that are ruining us.	0.70	-0.02
RWA08r	Gays and lesbians are just as healthy and moral as anybody else.	0.46	0.19
RWA09	The only way our country can get through the crisis ahead is to get back to our traditional values, put some tough leaders in power, and silence the troublemakers spreading bad ideas.	0.69	0.02
RWA10r	There is absolutely nothing wrong with nudist camps.	0.56	-0.12
RWA11r	Our country needs free thinkers who will have the courage to defy traditional ways, even if this upsets many people.	0.35	0.07
RWA12r	Everyone should have their own life-style, religious beliefs, and sexual preferences, even if it makes them different from everyone else.	0.29	0.25
RWA13	The "old-fashioned ways" and "old-fashioned values" still show the best way to live.	0.60	0.10
RWA14r	You have to admire those who challenged the law and the majority's view by protesting for women's abortion rights, for animal rights, or to abolish school prayer.	0.45	0.25
RWA15	What our country really needs is a strong, determined leader who will crush evil, and take us back to our true path.	0.63	-0.03

Table S1 continued

Item	Text	Factor1	Factor2
RWA16	God's laws about abortion, pornography, and marriage must be strictly followed before it is too late, and those who break them must be strongly punished.	0.62	-0.03
RWA17r	There is nothing wrong with premarital sexual intercourse.	0.59	-0.06
RWA18	Our country will be great if we honour the ways of our forefathers, do what the authorities tell us to do, and get rid of the "rotten apples" who are ruining everything.	0.63	0.09
RWA19r	There is no "ONE right way" to live life; everybody has to create their own way.	0.37	0.07
RWA20r	Homosexuals and feminists should be praised for being brave enough to defy "traditional family values."	0.18	0.31
RWA21	This country would work a lot better if certain groups of troublemakers would just shut up and accept their group's traditional place in society.	0.58	0.19
RWA22	There are many radical, immoral people in our country today, who are trying to ruin it for their own godless purposes, whom the authorities should put out of action.	0.58	-0.10
RWA23	What our country needs most is discipline, with everyone following our leaders in unity.	0.58	0.03
RWA24r	It's better to have trashy magazines and radical pamphlets in our communities than to let the government have the power to censor them.	0.41	-0.14

Table S1 continued

Item	Text	Factor1	Factor2
RWA25	The facts on crime, sexual immorality, and the recent public disorders all show we have to crack down harder on deviant groups and troublemakers if we are going to save our moral standards and preserve law and order.	0.57	-0.04
RWA26r	A lot of our rules regarding modesty and sexual behaviour are just customs which are not necessarily any better or holier than those which other people follow.	0.41	0.06
RWA27	The situation in our country is getting so serious, the strongest methods would be justified if they eliminated the troublemakers and got us back to our true path.	0.55	0.03
RWA28r	A "woman's place" should be wherever she wants to be. The days when women are submissive to their husbands and social conventions belong strictly in the past.	0.39	0.22
RWA29r	It is wonderful that young people today have greater freedom to protest against things they don't like, and to make their own "rules" to govern their behaviour.	0.33	0.24
RWA30	Once our government leaders give us the "go ahead," it will be the duty of every patriotic citizen to help stomp out the rot that is poisoning our country from within.	0.60	0.04
**SDO01**	**It's OK if some groups have more of a chance in life than others.**	**0.17**	**0.39**
**SDO02**	**Inferior groups should stay in their place.**	**0.32**	**0.36**
**SDO03**	**To get ahead in life, it is sometimes necessary to step on other groups.**	**0.11**	**0.40**

Table S1 continued

Item	Text	Factor1	Factor2
**SDO04r**	**We should have increased social equality.**	**-0.01**	**0.72**
**SDO05r**	**It would be good if groups could be equal.**	**-0.05**	**0.76**
**SDO06r**	**We should do what we can to equalise conditions for different groups.**	**0.00**	**0.78**
SDO07	Some groups of people are simply inferior to other groups.	0.36	0.33
SDO08	In getting what you want, it is sometimes necessary to use force against other groups.	0.14	0.38
SDO09	If certain groups stayed in their place, we would have fewer problems.	0.39	0.27
SDO10	It's probably a good thing that certain groups are at the top and other groups are at the bottom.	0.21	0.48
SDO11	Sometimes other groups must be kept in their place.	0.42	0.27
SDO12r	Group equality should be our ideal.	-0.08	0.68
SDO13r	All groups should be given an equal chance in life.	0.07	0.50
SDO14r	We would have fewer problems if we treated people more equally.	0.01	0.59
SDO15r	We should strive to make incomes as equal as possible.	-0.04	0.57
SDO16r	No one group should dominate in society.	0.15	0.36

Table S2

Self-report items from the New Zealand Attitudes and Values Study used in both Studies 1 and 2.

Item	Description / Text	Wave
SDO1	It is OK if some groups have more of a chance in life than others	10
SDO2	Inferior groups should stay in their place	10
SDO3	To get ahead in life, it is sometimes okay to step on other groups	10
SDO4 (reversed)	We should have increased social equality	10
SDO5 (reversed)	It would be good if groups could be equal	10
SDO6 (reversed)	We should do what we can to equalise conditions for different groups	10
RWA1	It is always better to trust the judgment of the proper authorities in government and religion than to listen to the noisy rabble-rousers in our society who are trying to create doubt in people's minds	10
RWA2	It would be best for everyone if the proper authorities censored magazines so that people could not get their hands on trashy and disgusting material	10
RWA3	Our country will be destroyed some day if we do not smash the perversions eating away at our moral fibre and traditional beliefs	10
RWA4 (reversed)	People should pay less attention to The Bible and other old traditional forms of religious guidance, and instead develop their own personal standards of what is moral and immoral	10

Table S2 continued

Item	Description / Text	Wave
RWA5 (reversed)	Atheists and others who have rebelled against established religions are no doubt every bit as good and virtuous as those who attend church regularly	10
RWA6 (reversed)	Some of the best people in our country are those who are challenging our government, criticizing religion, and ignoring the 'normal way' things are supposed to be done	10
Income redistribution	Redistributing money and wealth more evenly among a larger percentage of the people in New Zealand through heavy taxes on the rich	10
Environmental sacrifice	Are you willing to make sacrifices to your standard of living (e.g., accept higher prices, drive less, conserve energy) in order to protect the environment?	9
Support for Jobseeker Support	Increase payments for those receiving Jobseeker Support (formerly the Unemployment Benefit)	6
Support for Sole Parent Support	Increase payments for those receiving Sole Parent Support (formerly the Domestic Purposes Benefit)	6
Support for flat tax	A 'flat' tax rate (everyone pays the same percentage of tax on their income)	5
Income attribution	If incomes were more equal, people would be less motivated to work hard	10
Support for same-sex marriage	Same-sex marriage in NZ (The Marriage Amendment Act 2013)	9

Table S2 continued

Item	Description / Text	Wave
Support for euthanasia	Suppose a person has a painful incurable disease. Do you think that doctors should be allowed by law to end the patient's life if the patient requests it?	9
Support for abortion (any reason)	Legalized abortion for women, regardless of the reason	10
Support for abortion (specific reason)	Legalized abortion when the woman's life is endangered	10
Support for religious instruction	Including religious instruction in Christianity as part of the school curriculum	10
Race essentialism	To a large extent, a person's race biologically determines his or her abilities	10
Sexual prejudice (reversed)	I think that homosexuality should be accepted by society	10
Age	What is your date of birth?	10
Gender	What is your gender? (open-ended)	10
Ethnicity	Which ethnic group do you belong to? (NZ census question)	10
Education level	NZ Reg (0-10 education ordinal rank)	10
Socio-economic status	NZSEI13 (NZ Socio-economic index)	10
Local deprivation	Deprivation score 2013 (for Meshblock)	10
Religiosity	Do you identify with a religion and/or spiritual group?	10

Table S3

Percentage of participants correctly answering comprehension questions for tasks in Studies 1 and 2. For wording of comprehension questions, see survey materials on OSF: https://osf.io/dwx8g/

Wave	Game	Answered question correctly (%)
1	Stag Hunt Game	83
1	Trust Game	92
1	Public Goods Game	75
1	Dictator Game	98
1	Ultimatum Game	85
1	Third-Party Punishment Game	91
1	Second-Party Punishment Game	74
1	Stag Hunt Game with Punishment	85
2	BEAST	96
2	Rule Following Task	97

Table S4

Summary of the regression coefficients predicting the cooperation and punishment latent variables in the full structural equation model in Study 1.

DV	IV	Estimate	SE	z	p
Cooperation	SDO	-0.034	0.006	-6.189	.000
	RWA	-0.003	0.005	-0.559	.576
	Age	-0.001	0.000	-3.037	.002
	Gender (Male = 1)	0.009	0.010	0.944	.345
	Ethnicity (Pakeha)	0.010	0.012	0.775	.438
	Education	0.002	0.002	0.970	.332
	SES	-0.003	0.004	-0.713	.476
	Deprivation	0.012	0.006	1.903	.057
	Religious (Yes = 1)	0.005	0.010	0.473	.637
Punishment	SDO	0.005	0.002	3.467	.001
	RWA	-0.001	0.001	-0.951	.342
	Age	0.000	0.000	2.613	.009
	Gender (Male = 1)	-0.002	0.002	-0.680	.496
	Ethnicity (Pakeha)	0.003	0.003	0.860	.390
	Education	0.000	0.001	-0.319	.750
	SES	0.000	0.001	-0.371	.711
	Deprivation	0.001	0.002	0.654	.513
	Religious (Yes = 1)	0.002	0.003	0.868	.385

Table S5

Summary of the regression coefficients predicting preference for income redistribution (1-7) in the full structural equation model in Study 1. Full item: "Redistributing money and wealth more evenly among a larger percentage of the people in New Zealand through heavy taxes on the rich." Dependent variable is treated as ordinal.

IV	Estimate	SE	Lower 95% CI	Upper 95% CI	z	p
Cooperation	1.078	0.308	0.475	1.681	3.505	.000
Punishment	-2.674	1.242	-5.108	-0.240	-2.154	.031
Age	-0.006	0.003	-0.012	0.000	-1.951	.051
Gender (Male = 1)	-0.231	0.070	-0.369	-0.094	-3.299	.001
Ethnicity (Pakeha)	-0.206	0.091	-0.383	-0.028	-2.265	.024
Education	0.078	0.015	0.048	0.108	5.152	.000
SES	0.013	0.026	-0.038	0.064	0.497	.619
Deprivation	0.123	0.049	0.026	0.220	2.490	.013
Religious (Yes = 1)	-0.107	0.071	-0.246	0.033	-1.500	.133

Table S6

Summary of the regression coefficients predicting income attribution (1-7) in the full structural equation model in Study 1. Full item: "Income equality reduces motivation to work hard." Dependent variable is treated as ordinal.

IV	Estimate	SE	Lower 95% CI	Upper 95% CI	z	p
Cooperation	-1.333	0.308	-1.936	-0.730	-4.333	.000
Punishment	2.915	1.267	0.432	5.397	2.301	.021
Age	-0.008	0.003	-0.014	-0.002	-2.683	.007
Gender (Male = 1)	0.342	0.072	0.201	0.484	4.736	.000
Ethnicity (Pakeha)	-0.017	0.090	-0.192	0.158	-0.189	.850
Education	-0.045	0.015	-0.074	-0.016	-3.052	.002
SES	-0.024	0.027	-0.076	0.028	-0.907	.364
Deprivation	-0.132	0.049	-0.228	-0.035	-2.665	.008
Religious (Yes = 1)	0.062	0.073	-0.082	0.205	0.844	.399

Table S7

Summary of the regression coefficients predicting willingness to sacrifice for the environment (1-7) in the full structural equation model in Study 1. Full item: "Are you willing to make sacrifices to your standard of living (e.g., accept higher prices, drive less, conserve energy) in order to protect the environment?" Dependent variable is treated as ordinal.

IV	Estimate	SE	Lower 95% CI	Upper 95% CI	z	p
Cooperation	1.119	0.393	0.349	1.888	2.850	.004
Punishment	-2.611	1.390	-5.334	0.112	-1.879	.060
Age	-0.005	0.003	-0.011	0.002	-1.415	.157
Gender (Male = 1)	-0.285	0.078	-0.438	-0.131	-3.626	.000
Ethnicity (Pakeha)	-0.108	0.105	-0.315	0.099	-1.026	.305
Education	0.052	0.017	0.018	0.085	2.990	.003
SES	0.019	0.028	-0.036	0.074	0.678	.498
Deprivation	-0.069	0.052	-0.171	0.034	-1.315	.188
Religious (Yes = 1)	-0.280	0.077	-0.431	-0.130	-3.645	.000

Table S8

Summary of the regression coefficients predicting support for same-sex marriage (1-7) in the full structural equation model in Study 1. Full item: "Support for same-sex marriage in NZ (The Marriage Amendment Act 2013)." Dependent variable is treated as ordinal.

IV	Estimate	SE	Lower 95% CI	Upper 95% CI	z	p
Cooperation	0.399	0.412	-0.408	1.205	0.969	.333
Punishment	0.121	1.389	-2.601	2.843	0.087	.931
Age	-0.015	0.004	-0.022	-0.008	-4.067	.000
Gender (Male = 1)	-0.426	0.087	-0.597	-0.255	-4.883	.000
Ethnicity (Pakeha)	0.160	0.109	-0.055	0.374	1.460	.144
Education	0.052	0.018	0.017	0.088	2.886	.004
SES	0.026	0.031	-0.036	0.087	0.816	.414
Deprivation	-0.091	0.060	-0.207	0.026	-1.520	.128
Religious (Yes = 1)	-0.859	0.087	-1.029	-0.689	-9.899	.000

Table S9

Summary of the regression coefficients predicting support for euthanasia (1-7) in the full structural equation model in Study 1. Full item: "Suppose a person has a painful incurable disease. Do you think that doctors should be allowed by law to end the patient's life if the patient requests it?" Dependent variable is treated as ordinal.

IV	Estimate	SE	Lower 95% CI	Upper 95% CI	z	p
Cooperation	0.182	0.385	-0.572	0.936	0.472	.637
Punishment	0.924	1.335	-1.693	3.541	0.692	.489
Age	-0.007	0.003	-0.014	0.000	-1.999	.046
Gender (Male = 1)	-0.018	0.088	-0.191	0.154	-0.210	.833
Ethnicity (Pakeha)	0.107	0.108	-0.104	0.318	0.994	.320
Education	-0.014	0.019	-0.052	0.024	-0.727	.467
SES	-0.094	0.031	-0.155	-0.033	-3.027	.002
Deprivation	-0.033	0.055	-0.140	0.075	-0.592	.554
Religious (Yes = 1)	-0.795	0.082	-0.956	-0.635	-9.732	.000

Table S10

Summary of the regression coefficients predicting support for abortion (1-7) in the full structural equation model in Study 1. Full item: "Support for legalized abortion for women, regardless of the reason." Dependent variable is treated as ordinal.

IV	Estimate	SE	Lower 95% CI	Upper 95% CI	z	p
Cooperation	0.514	0.328	-0.130	1.157	1.564	.118
Punishment	1.048	1.187	-1.279	3.375	0.883	.377
Age	-0.014	0.003	-0.021	-0.008	-4.269	.000
Gender (Male = 1)	-0.204	0.078	-0.356	-0.052	-2.636	.008
Ethnicity (Pakeha)	-0.005	0.099	-0.200	0.190	-0.050	.960
Education	0.052	0.017	0.018	0.085	2.987	.003
SES	0.023	0.029	-0.034	0.081	0.800	.424
Deprivation	-0.096	0.051	-0.197	0.005	-1.867	.062
Religious (Yes = 1)	-0.853	0.077	-1.004	-0.702	-11.081	.000

Table S11

Summary of the regression coefficients predicting support for increased payments for those receiving Jobseeker Support (1-7) in the full structural equation model in Study 1. Full item: "Increase payments for those receiving Jobseeker Support (formerly the Unemployment Benefit)." Dependent variable is treated as ordinal.

IV	Estimate	SE	Lower 95% CI	Upper 95% CI	z	p
Cooperation	1.306	0.343	0.633	1.979	3.806	.000
Punishment	-0.887	1.207	-3.252	1.478	-0.735	.462
Age	0.003	0.003	-0.003	0.009	1.048	.295
Gender (Male = 1)	-0.085	0.075	-0.232	0.061	-1.145	.252
Ethnicity (Pakeha)	-0.049	0.093	-0.232	0.134	-0.527	.598
Education	0.060	0.015	0.030	0.089	3.933	.000
SES	-0.008	0.026	-0.059	0.044	-0.301	.763
Deprivation	0.145	0.052	0.042	0.247	2.764	.006
Religious (Yes = 1)	-0.081	0.075	-0.228	0.066	-1.082	.279

Table S12

Summary of the regression coefficients predicting support for increased payments for those receiving Sole Parent Support (1-7) in the full structural equation model in Study 1. Full item: "Increase payments for those receiving Sole Parent Support (formerly the Domestic Purposes Benefit)." Dependent variable is treated as ordinal.

IV	Estimate	SE	Lower 95% CI	Upper 95% CI	z	p
Cooperation	1.404	0.347	0.724	2.084	4.048	.000
Punishment	-1.343	1.225	-3.744	1.057	-1.097	.273
Age	-0.006	0.003	-0.012	0.000	-1.839	.066
Gender (Male = 1)	-0.180	0.074	-0.326	-0.034	-2.413	.016
Ethnicity (Pakeha)	-0.017	0.095	-0.204	0.169	-0.182	.856
Education	0.063	0.015	0.033	0.094	4.098	.000
SES	0.004	0.026	-0.048	0.055	0.144	.885
Deprivation	0.150	0.051	0.049	0.251	2.913	.004
Religious (Yes = 1)	-0.103	0.075	-0.249	0.043	-1.382	.167

Table S13

Summary of the regression coefficients predicting support for a flat tax (1-7) in the full structural equation model in Study 1. Full item: “Support for a ‘flat’ tax rate (everyone pays the same percentage of tax on their income).” Dependent variable is treated as ordinal.

IV	Estimate	SE	Lower 95% CI	Upper 95% CI	z	p
Cooperation	-0.697	0.324	-1.333	-0.062	-2.150	.032
Punishment	0.317	1.152	-1.941	2.574	0.275	.783
Age	0.001	0.003	-0.004	0.007	0.447	.655
Gender (Male = 1)	0.147	0.073	0.003	0.290	2.007	.045
Ethnicity (Pakeha)	-0.041	0.096	-0.229	0.147	-0.427	.670
Education	-0.055	0.016	-0.087	-0.022	-3.316	.001
SES	-0.037	0.027	-0.091	0.016	-1.373	.170
Deprivation	0.022	0.050	-0.076	0.121	0.445	.656
Religious (Yes = 1)	0.135	0.075	-0.012	0.282	1.794	.073

Table S14

Test-retest reliability for the economic games from previous work[47] *across Studies 1 and 2. Correlations are Spearman's rank correlations (all* $p < .050$*). Intra-class correlations are two-way single score agreements across the two studies (all* $p < .050$*).*

Game	Correlation	ICC
Trust Game (Give)	0.24	0.24
Trust Game (Return)	0.28	0.28
Public Goods Game	0.29	0.30
Dictator Game	0.43	0.43
Ultimatum Game (Offer)	0.23	0.14
Ultimatum Game (Minimum Acceptable Offer)	0.41	0.40
Third-Party Punishment Game (Steal)	0.20	0.20
Third-Party Punishment Game (Punish)	0.23	0.23
Second-Party Punishment Game (Transfer)	0.14	0.13
Second-Party Punishment Game (Punish Cooperator)	0.47	0.40
Second-Party Punishment Game (Punish Defector)	0.24	0.24

Table S15

Summary of the fixed effects from the Bayesian multilevel logistic regression model predicting rule following in Study 2.

IV	Estimate	SE	Lower 95% CI	Upper 95% CI
Intercept	1.848	0.374	1.118	2.573
Round number	0.407	0.325	-0.219	1.042
RWA	0.080	0.249	-0.422	0.575
SDO	-0.659	0.276	-1.192	-0.099
Gender (Male = 1)	-0.184	0.400	-0.959	0.613
Age	0.011	0.025	-0.038	0.059
Deprivation	0.358	0.324	-0.286	0.978
Ethnicity (Maori)	0.669	0.446	-0.214	1.526
Ethnicity (Pacific)	0.184	0.492	-0.792	1.145
Ethnicity (Pakeha)	1.063	0.388	0.297	1.809
Education	-0.226	0.128	-0.477	0.024
SES	-0.021	0.210	-0.435	0.385

Table S16

Summary of the fixed effects from the Bayesian multilevel beta regression model predicting BEAST scores in Study 2.

IV	Estimate	SE	Lower 95% CI	Upper 95% CI
Intercept	-0.715	0.315	-1.329	-0.105
RWA	0.146	0.046	0.053	0.237
SDO	0.058	0.055	-0.049	0.166
Gender (Male = 1)	0.024	0.105	-0.179	0.227
Age	-0.001	0.004	-0.008	0.007
Deprivation	0.038	0.067	-0.094	0.170
Ethnicity (Maori)	-0.451	0.306	-1.045	0.160
Ethnicity (Pacific)	-0.145	0.427	-0.994	0.695
Ethnicity (Pakeha)	-0.372	0.280	-0.908	0.174
Education	-0.007	0.021	-0.048	0.034
SES	0.005	0.037	-0.064	0.079

Table S17

Summary of the regression coefficients predicting support for same-sex marriage (1-7) in the Bayesian ordinal regression model in Study 2. Full item: Support for "same-sex marriage in NZ (The Marriage Amendment Act 2013)."

IV	Estimate	SE	Lower 95% CI	Upper 95% CI
Cutpoint 1	-2.695	0.364	-3.415	-1.981
Cutpoint 2	-2.214	0.350	-2.929	-1.522
Cutpoint 3	-1.931	0.343	-2.631	-1.244
Cutpoint 4	-1.387	0.337	-2.061	-0.714
Cutpoint 5	-0.992	0.333	-1.670	-0.331
Cutpoint 6	-0.270	0.328	-0.935	0.378
BEAST score	-0.339	0.331	-1.001	0.301
Gender (Male = 1)	-0.637	0.176	-0.977	-0.300
Age	-0.033	0.007	-0.047	-0.019
Deprivation	-0.263	0.121	-0.502	-0.023
Ethnicity (Maori)	0.495	0.330	-0.147	1.132
Ethnicity (Pacific)	-0.091	0.431	-0.931	0.752
Ethnicity (Pakeha)	0.395	0.306	-0.209	0.993
Education	0.059	0.039	-0.018	0.136
SES	0.060	0.066	-0.067	0.187

Table S18

Summary of the regression coefficients predicting support for euthanasia (1-7) in the Bayesian ordinal regression model in Study 2. Full item: "Suppose a person has a painful incurable disease. Do you think that doctors should be allowed by law to end the patient's life if the patient requests it?"

IV	Estimate	SE	Lower 95% CI	Upper 95% CI
Cutpoint 1	-2.766	0.357	-3.455	-2.043
Cutpoint 2	-2.351	0.346	-3.015	-1.655
Cutpoint 3	-2.113	0.342	-2.764	-1.428
Cutpoint 4	-1.512	0.330	-2.147	-0.862
Cutpoint 5	-0.974	0.324	-1.587	-0.327
Cutpoint 6	-0.104	0.321	-0.714	0.545
BEAST score	-0.532	0.331	-1.176	0.107
Gender (Male = 1)	-0.217	0.163	-0.547	0.103
Age	-0.019	0.007	-0.033	-0.005
Deprivation	-0.108	0.121	-0.344	0.127
Ethnicity (Maori)	0.210	0.314	-0.396	0.807
Ethnicity (Pacific)	-0.061	0.408	-0.858	0.756
Ethnicity (Pakeha)	0.366	0.297	-0.206	0.977
Education	-0.057	0.039	-0.133	0.022
SES	-0.112	0.066	-0.245	0.013

Table S19

Summary of the regression coefficients predicting support for abortion for any reason (1-7) in the Bayesian ordinal regression model in Study 2. Full item: Support for "legalized abortion for women, regardless of the reason."

IV	Estimate	SE	Lower 95% CI	Upper 95% CI
Cutpoint 1	-2.984	0.350	-3.674	-2.307
Cutpoint 2	-2.270	0.329	-2.919	-1.618
Cutpoint 3	-1.908	0.322	-2.537	-1.266
Cutpoint 4	-1.161	0.316	-1.772	-0.532
Cutpoint 5	-0.621	0.315	-1.232	0.000
Cutpoint 6	0.167	0.315	-0.451	0.783
BEAST score	-0.759	0.325	-1.406	-0.116
Gender (Male = 1)	-0.284	0.153	-0.580	0.022
Age	-0.032	0.007	-0.045	-0.019
Deprivation	-0.099	0.108	-0.309	0.112
Ethnicity (Maori)	0.430	0.310	-0.175	1.036
Ethnicity (Pacific)	0.320	0.401	-0.443	1.145
Ethnicity (Pakeha)	0.439	0.289	-0.133	1.013
Education	0.067	0.036	-0.002	0.137
SES	-0.009	0.061	-0.128	0.109

Table S20

Summary of the regression coefficients predicting support for abortion when the woman's life is endangered (1-7) in the Bayesian ordinal regression model in Study 2. Full item: Support for "legalized abortion when the woman's life is endangered."

IV	Estimate	SE	Lower 95% CI	Upper 95% CI
Cutpoint 1	-4.263	0.464	-5.198	-3.381
Cutpoint 2	-3.782	0.411	-4.592	-2.998
Cutpoint 3	-3.525	0.394	-4.296	-2.772
Cutpoint 4	-2.674	0.359	-3.398	-1.994
Cutpoint 5	-2.202	0.343	-2.882	-1.548
Cutpoint 6	-1.155	0.331	-1.841	-0.528
BEAST score	-0.759	0.361	-1.462	-0.052
Gender (Male = 1)	-0.362	0.186	-0.715	0.002
Age	-0.010	0.008	-0.026	0.005
Deprivation	-0.158	0.130	-0.417	0.094
Ethnicity (Maori)	0.096	0.329	-0.552	0.760
Ethnicity (Pacific)	-0.272	0.424	-1.061	0.595
Ethnicity (Pakeha)	0.345	0.300	-0.257	0.912
Education	0.092	0.043	0.009	0.177
SES	-0.095	0.074	-0.239	0.049

Table S21

Summary of the regression coefficients predicting support for religious education in schools (1-7) in the Bayesian ordinal regression model in Study 2. Full item: Support for "including religious instruction in Christianity as part of the school curriculum."

IV	Estimate	SE	Lower 95% CI	Upper 95% CI
Cutpoint 1	-0.772	0.296	-1.348	-0.190
Cutpoint 2	-0.281	0.294	-0.853	0.307
Cutpoint 3	0.192	0.294	-0.391	0.773
Cutpoint 4	1.075	0.297	0.508	1.664
Cutpoint 5	1.854	0.309	1.260	2.465
Cutpoint 6	2.580	0.336	1.938	3.256
BEAST score	0.749	0.316	0.122	1.361
Gender (Male = 1)	-0.302	0.155	-0.603	0.002
Age	0.017	0.006	0.005	0.029
Deprivation	-0.112	0.107	-0.323	0.099
Ethnicity (Maori)	-0.104	0.298	-0.691	0.492
Ethnicity (Pacific)	0.138	0.378	-0.614	0.867
Ethnicity (Pakeha)	-0.558	0.271	-1.081	-0.014
Education	-0.066	0.034	-0.132	0.000
SES	0.006	0.060	-0.111	0.124

Table S22

Summary of the regression coefficients predicting race essentialism (1-7) in the Bayesian ordinal regression model in Study 2. Full item: "To a large extent, a person's race biologically determines his or her abilities."

IV	Estimate	SE	Lower 95% CI	Upper 95% CI
Cutpoint 1	0.308	0.304	-0.289	0.905
Cutpoint 2	1.310	0.307	0.714	1.901
Cutpoint 3	1.978	0.313	1.370	2.593
Cutpoint 4	2.657	0.326	2.027	3.297
Cutpoint 5	3.547	0.369	2.842	4.291
Cutpoint 6	4.825	0.526	3.857	5.911
BEAST score	0.618	0.334	-0.035	1.278
Gender (Male = 1)	0.361	0.165	0.038	0.667
Age	-0.003	0.007	-0.016	0.010
Deprivation	0.098	0.111	-0.117	0.310
Ethnicity (Maori)	-0.132	0.307	-0.741	0.481
Ethnicity (Pacific)	-0.336	0.406	-1.136	0.457
Ethnicity (Pakeha)	-0.232	0.276	-0.769	0.318
Education	-0.076	0.037	-0.148	-0.002
SES	0.011	0.063	-0.114	0.132

Table S23

Summary of the regression coefficients predicting prejudice against homosexuality (1-7) in the Bayesian ordinal regression model in Study 2. Full item: "I think that homosexuality should be accepted by society" (reversed).

IV	Estimate	SE	Lower 95% CI	Upper 95% CI
Cutpoint 1	0.169	0.307	-0.417	0.782
Cutpoint 2	1.085	0.312	0.496	1.695
Cutpoint 3	1.490	0.318	0.890	2.119
Cutpoint 4	2.326	0.330	1.705	2.986
Cutpoint 5	2.982	0.355	2.306	3.684
Cutpoint 6	3.365	0.375	2.655	4.105
BEAST score	0.642	0.330	0.014	1.294
Gender (Male = 1)	0.612	0.166	0.297	0.939
Age	0.030	0.007	0.016	0.044
Deprivation	0.131	0.114	-0.093	0.347
Ethnicity (Maori)	-0.399	0.315	-1.013	0.213
Ethnicity (Pacific)	0.031	0.418	-0.776	0.834
Ethnicity (Pakeha)	-0.614	0.289	-1.167	-0.050
Education	-0.077	0.038	-0.151	-0.003
SES	-0.080	0.066	-0.211	0.047
